# A new strategy for the computer-assisted development of reversed-phase liquid chromatography separation methods of unknown sample mixtures

**DOI:** 10.1007/s00216-021-03538-7

**Published:** 2021-08-18

**Authors:** R. Cela, S. Triñanes, C. Cobas

**Affiliations:** 1 Analytical Chemistry Laboratory, Research Institute for Chemical and Biological Analyses, University of Santiago de Compostela, Santiago, Spain; 2 Mestrelab Research S.L. Santiago de Compostela, Santiago, Spain

**Keywords:** Computer simulation, Liquid chromatography, Reversed phase, MCR-ALS, Optimization

## Abstract

A new strategy for the computer-assisted methods development in the reversed-phase liquid chromatographic separations of unknown sample mixtures has been developed using the latent spectral information in chromatogram raw data files of appropriately designed experiments, rather than resorting to elemental information functions (e.g., the number of peaks in chromatograms or similar criteria). The strategy developed allows unification of the approach for samples of both known and unknown composition and, thus, provide a general strategy for computer-aided tools in the chromatography laboratory. The operation principle of this strategy departs from extracting the spectra of components in the mixture chromatograms by resorting to multivariate curve resolution-alternating least squares (MCR-ALS). This technique allows the estimation of the true spectra for the individual components except when they have identical spectra or are fully overlapped. Thus, a convenient experimental design will try to perform separations of the sample mixture having at least partial resolution of components in some runs. This will allow estimating the spectra of components and, then, assign these components to the peaks in each run chromatogram. In this way, a retention model can be built for each component so computerized optimization process can be developed to provide the chromatographer with the best possible separation programs. Following this approach, strategies for sample mixtures of known and unknown composition are only different in the need of an initial spectrum discovery process for unknown mixtures and therefore a real general approach for the computer-assisted LC methods development is now available for the first time.

**Supplementary Information:**

The online version contains supplementary material available at 10.1007/s00216-021-03538-7.

## Introduction

Computerized method development in high-performance liquid chromatography (HPLC) emerged as soon as the computers entered the analytical laboratory in the past 1970s. By this time, LC practitioners have clearly realized the power of the technique and, also the difficulties to develop good elution programs for real-world samples using the classical trial-and-error approach, due to the many variables that must be taken into account and the slowness of the trials. In general, it was accepted that the development of new separation procedures required time, dedication, and expertise of chromatographers, so the possibility of having a computerized tool taking care of experimental design, instrument’s control, data analysis, and decision-making appeared as a highly desirable goal [1]. The first commercial systems aimed to these objectives were quite ephemeral [2, 3] and many other proposals remain as pure academic studies [4, 5]. Only a few dedicated software packages that work independently of instrument’s manufacturers have remained in the market after more than 40 years with periodical updates, being Drylab [6], probably, the best known and more representative example of this assertion. The majority of these applications have evolved to provide an efficient communication with the instruments of the main LC manufacturers, thus approaching the original goal as mentioned above. However, a major limitation has remained from the outset: the need to have the knowledge about the components mixed in the sample.

Restricting our discussion to reversed-phase LC, which continues to be the most preferred LC mode, LC computer-assisted methods development (CAMD) program builds a model of the retention of the sample components as a function of the chromatographic column selected, the mobile phase, and other experimental variables such as the temperature, pH, or the nature of the modifier. An experimental plan is designed to run the calibrating elutions needed for the model while reducing the number of runs as much as possible. The obtained chromatograms are processed to assign the retention of each component in each run. The obtained retention times are processed to build the retention model using any of the theoretical models available [7]. The so-called linear solvent strength model (LSSM) [8] is widely used for these purposes. This model is often expressed by the simple equation:

1 $$ \log k=\log {k}_0- S\varphi $$

where *ϕ* is the volume fraction of the modifier in the mobile phase (% B expressed in decimal form), *S* a constant for a given compound, and log *k*_0_ the theorical value of the retention factor corresponding to an isocratic elution using pure water as mobile phase. Other models [9, 10] perform better for analytes that deviate from the linear relationship. This retention model may now be applied in simulation processes to explore and optimize elution programs in the search for a convenient separation of the sample components.

As stated before, to build the model, each component in the sample should be identified accurately in all calibrating chromatograms, so we need to know beforehand how many components form the mixture and how to identify each one. The exact composition of the mixture should be accurately known before starting any CAMD process. Spectral characteristics of mixture components may be used to locate each component in the chromatograms and, with some practical limitations, the peak areas can help when overlapping occurs. Many times not all mixture components are of interest. However, this does not mean that we do not need to know about their existence and their characteristics because all components must be modelled to optimize the separation of the components of interest. Otherwise, unmodelled interferents may invalidate the optimization process (frequently based on the so-called resolution maps, which measure the critical Rs in chromatograms as a measure of the separation feasibility). In real-life problems, this limitation is important because, very often, we do not have all the needed information about the samples, and thus, the CAMD tool cannot be applied or must be applied under suboptimal conditions.

The first practical approach to deal with separations of unknown composition mixtures was described by Krull and coworkers [11] in 2008–2009, coining the terms of “named” and “unnamed” peaks and proposing the concept of “trend responses.” Unnamed peak problems are separation problems where the number of peaks and/or the nature of some, or all, components in the mixtures to be separated is not known, and thus, the peaks cannot be tracked along the model calibration chromatograms, and thus, no retention model can be built. Changes in selectivity caused by modifications in the LC operational variables are the key to solving separation problems but that changes cannot be elucidated if the particular position of each peak in the chromatograms cannot be tracked. Thus, Krull and coworkers proposed an essentially different approach using the information that can be derived directly and easily from the chromatograms (e.g., the number of peaks readily visible in each chromatogram, the number of peak pairs exhibiting good resolution or good shape and symmetry in chromatograms; the peak having the largest area; the time needed to elute all apparent components in the sample, or to elute the first peak). The commercial software Fusion QbD [12] is the practical tool using these concepts.

Although the proposal was appealing, the practical application exhibits critical limitations even for mixtures of low-medium complexity, especially when desirable changes in selectivity appear during the screening runs. Some of these limitations have been demonstrated with real examples [1] so the CAMD for unnamed peak problems remain still an unresolved problem except for the simpler situations.

Recently, the application of multivariate curve resolution-alternating least squares (MCR-ALS) technique [13, 14] to the resolution of overlapped signals in chromatography [15, 16] and the assessment of peak purity [17] has received considerable attention. MCR-ALS allows evaluating the number of components overlapped in a chromatographic peak and, if the chromatograms are conveniently arranged, quantitating these components.

In an attempt to overcome the essential limitation of the existing CAMD tools when dealing with mixtures of unknown composition, an alternative strategy has been developed that makes use of the qualitative spectral information latent in the raw data files of the screening chromatographic runs, which can be extracted by the use of MCR-ALS. That information is used to evaluate the number of components in the mixture and to assign their positions in the chromatograms. In this way, the retention model can be built and applied to develop further optimization process by computer simulation. A far as we are aware, this is the first strategy that can deal efficiently with unknown composition mixtures of medium-high complexity. Moreover, the proposed strategy unifies the way both types of separation problems (named and unnamed peaks) are processed and therefore breaks the classical barrier established by the knowledge about the sample composition. This means that the proposed strategy serves all the original objectives of CAMD providing a general solution to the chemometrical development of reversed-phase LC separations.

## Theory

The proposed algorithm to modelling the retention of peaks in samples of unknown composition takes the following steps:

1. Select one or several chromatographic columns and mobile phases to screen the mixture.
2. Obtain a series of chromatogram runs for the mixture according to a convenient experimental design aimed at revealing the selectivity differences of the tested columns and mobile phases to the sample components.
3. Process the chromatogram files to define peaks or peak cluster’s locations.
4. Systematically apply the MCR-ALS algorithm to estimate the number of components in each peak or peak cluster in each chromatogram and their spectral and elution profile characteristics.
5. Complete the processing of each chromatogram to produce a list of estimated components in the sample mixture as seen in that chromatogram and categorize the discovered components corresponding to pure or mixed components in peaks.
6. Correlate all discovered chromatogram components in the experiments to generate a set of common components in the sample. If needed, the user participates now to verify the component’s assignation.
7. Take the retention of each component in each chromatogram to produce a retention model using the linear solvent strength model (thus restricting the tool for reversed-phase separations).
8. Use the retention model obtained to simulate separations in different elution conditions using an efficient optimization engine.
9. Take the optimized results, if satisfactory, and test experimentally the obtained separation program evaluating the robustness of the separation.

Because the novelty of the proposed tool consists essentially in the strategical combination of several chemometrical techniques and some of these steps adopt algorithmic solutions that have been extensively described and tested, the following paragraphs will only consider in detail those that are applied for the first time in the context of the computer-assisted methods development in LC.

### Steps 1 and 2. Experimental design

The number of columns and/or mobile phases to be considered in the screening process is clearly dependent on the information available about the mixture composition. For unknown mixtures, there is no other option that tests several columns exhibiting different selectivity and possibly several organic modifiers in the mobile phase. For partially unknown mixtures, the number of columns to be tested can be reduced considerably. The use of available tools that use the chemical and structural properties of the known mixture components allows reducing significantly the screening experimental work and selecting the more promising column and solvent selectivities [7]. On the other side, the objective in the experimental design is twofold. It should be able to provide the necessary data to build the retention model, and it should reveal as much as possible the differences in selectivity for the mixture components. The ability of the MCR-ALS algorithm proposed in step 4 to discover the component’s spectra and profiles may be hindered if components eluted exactly at the same retention time or if their spectra are identical and components elute highly or fully overlapped. Thus, the objective is to achieve, even if only, a partial separation of unresolved components in some of the runs. In that case, the spectra of components could be resolved in such runs, and then, in step 6, these components will be assigned in runs where they cannot be discovered initially. A third condition to be imposed to the experimental design, in order to accommodate it in a practical tool, is restricting the number of runs to a minimum. In our case, highly efficient Hoke D6 design matrices [18] have been adopted for the experimental design. These matrices are highly economic, requiring 2, 6, 8, and 18 runs for 1 to 4 experimental variables, and allow estimating second-order regression models with excellent accuracy. Experimental factors such as the proportion of modifier, the gradient steepness, temperature, and less frequently pH and the nature of modifier and/or the proportions of binary modifiers can be considered while keeping the number of screening runs reasonable. To accommodate the use of such design matrices to the LSS model, at least two different levels of the gradient time in a ratio 3:1 or higher should be adopted. Since the discovery algorithm will be based on spectral profiles for components, some variables, such as the pH, that frequently alter the UV spectrum of components are excluded if only such detectors are used. In most real-life problems, 4–8 runs are sufficient to develop the strategy. In addition, at least a couple of test points are recommended to validate the results. Therefore, 6–10 runs are needed usually in this strategy.

### Step 3. Raw data chromatogram processing

The principle of the proposed strategy considers that, although we do not have information about the nature or the spectral characteristics of the mixture components, this information is latent in the raw data files of the chromatographic runs provided we have used detectors giving second-order data formats (e.g., DAD-UV or full scan MS). Thus, the objective should be discovering and extract this spectral information from such raw data files. One of the most efficient procedures to do so is MCR-ALS (see step 4) [14]. This technique could be applied to process the complete chromatogram as shown by Monago et al. [19]. However, in general, the procedures adopted to process chromatographic signals by means of MCR-ALS assume as objective the quantification of one or several components in the sample that elute not fully resolved, and thus, the runs will consider calibration samples having different amounts of those components but the same elution program. On the contrary, for LC screening and method development, the sample composition is always the same, but the elution programs are changing as many times as rows are in the design matrix, so each chromatogram can be considered unique. Thus, our approach was based on considering small data sections in chromatograms separated by baseline signals. These sections, or peak clusters, may contain one or several components but, in any case, can be easily processed by the MCR-ALS algorithms described in the literature [20, 21]. Consequently, this second step in the strategy consists in obtaining the second derivative of the chromatographic signal and extract sections of the chromatogram delimited by baseline points. Then, inspect minima in the chromatogram profile to separate peak clusters into more simple units. To ensure that all component signals are detected, the maxplot (if a UV detector is used) and/or the TIC (when using MS detectors) are processed.

### Step 4. Multivariate curve resolution-alternating least squares (MCR-ALS)

The MCR term describes a family of algorithms for the assessment of the underlying contributions of the individual components in a mixture when this mixture is recorded as a data set. Any analytical procedure yielding linearly additive responses (e.g., DAD-UV or full scan MSD) will provide data sets appropriated to MCR. The scheme in Figure S1 (see Supplementary Information, ESM), adapted from [13], describes how MCR extracts the latent information in these data sets. Values of the X matrix correspond to detector response at any particular channel (e.g., the absorbance at the *j*th wavelength). This response has been produced because each of the N components in the mixture has a certain *c*_N_ concentration and a characteristic sensitivity factor (e.g., molar absorptivity for UV detectors) *s*_jn_. Thus, the data set (or sections of the data set as each peak cluster) can be decomposed into two informative matrices: C (the component’s concentration in general, or the component’s concentration profiles in our case) and S (the component’s spectra matrix), plus a residual’s matrix representing the error contribution to measurements. This is a bilinear model that can be represented as in ESM Figure S1.

To obtain both spectra and profiles for the matrix X, we need a starting point that can be estimated by several approaches derived from single value decomposition (SVD) analysis [22], such as resolving factor analysis [23] (RFA). Alternatively, other procedures such as the alternating least squares (ALS) give directly estimates of the C and S^T^ matrices by implementing the iterative calculation of these matrices from an initial estimate of either the concentration or the spectra matrix. The initial estimate for the spectral matrix produced by the simple-to-use self-modelling mixture analysis (SIMPLISMA) algorithm [24] performs quite efficiently [25] and was the approach adopted in our case.

MCR is affected by ambiguity although a unique solution is highly desirable in practice. To deal with MCR inherent ambiguities, some constraints (non-negativity, unimodality, selectivity, and closure) may be implemented into the algorithms. The adoption of these practical constraints is based on the theorems developed by Manne [26] and the characteristics of the analytical signals in the data set. Recently, some tools have been developed to help MCR practitioners in handling and evaluating the effects of these ambiguities [27]. Non-negativity and unimodality were applied in our case. Both constraints that restrict rotational ambiguity are easily understood for LC signals although frequently negative signals in baseline regions can be obtained due to noise or inadequate auto zeroing, so raw data signals have to be preprocessed to ensure positive values. Unimodality, meaning that pure components would have unimodal profiles, is a common characteristic of chromatographic signals.

**Fig. 1:**
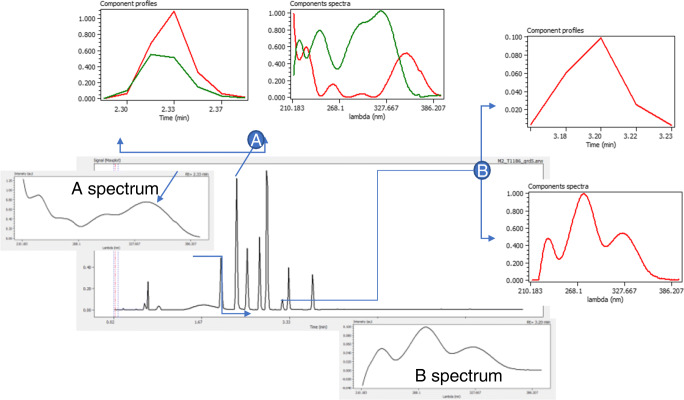
Extraction of components from a DAD-UV maxplot chromatogram peaks using MCR-ALS . Peak A represents a situation with strongly overlapping components. Peak B corresponds to a single component in the peak. Spectra plotted in black color correspond to actual spectra of both peaks as taken in the peak apex. Red and green plots correspond to profiles and spectra of the extracted components

The scheme in Fig. 1 represents the way peak clusters are processed to extract the components from raw data signals produced by a DAD-UV detector. Two peaks in the maxplot have been focused to show the process. In the first one (A), the MCR-ALS algorithm clearly indicates the presence of two components strongly overlapped. The spectrum recorded at the peak apex allows recognition of the sum of the signals of the two components. For peak B, only a pure component is extracted and the spectrum at the apex fits confirms this result. See that for peak B, the differences between the extracted and the actual spectra are mainly attributable to the negative values appearing in the actual spectrum that has been eliminated through the non-negativity constraint processing imposed to the MCR-ALS. Figure S2 (see ESM) shows a simplified flow chart of the code used to perform these processes, assuming that both DAD-UV and MS signals have been registered.

### Steps 5 and 6

The result of the whole process exemplified in Fig. 1 are two sets of component’s spectra for each chromatogram. One for pure components and the other for components that eluted overlapped in the chromatograms. Now, the component sets for all screening runs are combined. Some pure components possibly have been eluted as such in several or even all chromatograms. Others possibly have appeared pure only in a few chromatograms and some others have been eluted always overlapped with one or several components. Thus, a correlation analysis is done using the extracted component’s spectra to form two manageable sets of spectra as shown in the flow chart in Figure S3 (see ESM). The first component set join up the components that have been eluted as a pure component at least in one chromatogram and are uncorrelated to any other pure component and to any other component in the same run, so a set of unique spectra forms the first component’s set. Obviously, the second set is formed by the remaining spectra. Now, the set of pure components is compared (correlated) with all extracted components in each chromatogram, so the corresponding components will be assigned to peaks, irrespective of whether they elute pure or overlapping. Then, after excluding the assigned components in the overall list, the second set of component’s spectra is also submitted to a correlation analysis with the spectra of not assigned components in the list. The flow chart in Figure S3 (see ESM) shows a schematic view of these processes.

The basic hypothesis in this procedure is first to assume that using an experimental design that efficiently explores the operational variables any component in the mixture will have a non-negligible probability of eluting pure, or at least be partially resolved from other components, in some chromatograms, so the probability of having a consistent set of pure components formed by the majority of the sample components becomes high. Secondly, if MCR-ALS provides good estimates of component spectra both for pure eluting ones and for those that eluted overlapped, the correlation analysis will provide an accurate approach to the real composition of the sample. Of course, the MCR-ALS procedure may suffer from overfitting as any other regression approach and produce false positive components, in many cases caused by noisy signals. This means that for samples of medium-high complexity, it is often necessary to have a process of revision and cleaning of these false positives before accepting the retention model. Evidently, this process requires the participation of the chromatographer, and thus, the availability of helping tools is highly desirable in this process. Several of these helping tools have been developed and will be shown in the “Results and discussion” section.

### Step 7

Once the sample components have been discovered and false positives filtered out, a retention model of the sample components can be built. Because the developed system is assumed to be limited to reversed-phase separations, the linear solvent strength model developed by Snyder and coworkers [8] can be applied to build this retention model while keeping the number of screening experiments to a minimum. A standard partial least squares (PLS) regression was used to obtain the retention equation for each component in the sample. The diagnostics applied to that model, and especially the magnitude of prediction errors for validation runs, help in assessing the reliability of the discovery process developed in steps 5 and 6, because bad assignments of components to peaks will produce large residuals in the PLS regression model for that run. Thus, the assessment of the PLS model built for each peak is developed simultaneously with the false positive filtering process described in the above steps. The “Results and discussion” section will show examples of these processes.

### Steps 8 and 9

Once a satisfactory retention model has been calculated, this model can be used to simulate any kind of elution program profiles and assess the possibilities of separating the components of the sample. The procedures to develop the computer-simulated optimization of the separation of sample components follow the developments made in the steps model developed by Cela et al. in the 1980s [5]. In short, this model assumes that any elution profile can be simulated by a series of small isocratic steps that follow the overall profile needed. Retention in these isocratic steps is calculated from the retention model and the optimization process itself takes as variables the operational parameters selected by the chromatographer (e.g., the temperature) and the parameters of the elution profile. The optimization engine used to find the optimum separation program is an in-house implemented differential evolution algorithm [28] that may deal with uni- and multi-objective [29, 30] optimization processes. This algorithm provides several advantages as compared to regular genetic algorithms used in previous studies [31] both in convergence speed, population size, and calculation speed, so it is possible to produce optimized results in a few minutes even for rather complex samples. The multi-objective Pareto approach [32] is the recommended option as will be explained in the “Results and discussion” section, although more classical approaches are also available.

### Step 10

The last step in the process is the experimental verification of the optimal solution adopted through the CAMD process and the assessment of the elution robustness. As usual, this step may be developed by using a Plackett-Burman design [33] and evaluating the results. Also, the computerized tool can be applied to help in assessing the potential robustness of the chosen solution. In our case, the approach recently published by Triñanes et al. [34] is recommended without excluding the need of a final experimental verification of the optimal solution adopted and its real robustness.

## Experimental

### Chemicals

To evaluate the proposed strategy, two mixtures of polyphenolic compounds were prepared. The first one contained ten compounds and the second one fifteen. The standards used to build these mixtures (4-hydroxybenzoic, 3,4-dihydroxybenzoic, 2,5-dihydroxybenzoic, 3,4-dimethoxybenzoic, 2-hydroxycinnamic, 4-hydroxycinnamic, 3,4-dihydroxycinnamic, 3-caffeoylquinic acid, 4-hydroxy-3,5-dimethoxybenzaldehyde, 2,4-dimethoxybenzaldehyde, 2,5-dihydroxybenzaldehyde, 3-hydroxybenzaldehyde, 4-hydroxybenzaldehyde, 4-hydroxy-3-methoxybenzaldehyde, 3,4,5-trimethoxybenzaldehyde, 4-hydroxy-coumarin, 7-hydroxy-coumarin, and 6,7-dihydroxycoumarin) were supplied by Sigma-Aldrich (Merck Life Science, Madrid, Spain).

### Equipment

Chromatographic separations were developed using three different columns: a 75 × 4.6-mm, 5-μm particles Xbridge C18 and a 50 × 3.0-mm, 2.7-μm particles Cortecs C18, both supplied by Waters Cromatogafía (Cerdanyola del Valles, Spain), and a 50 × 4.6-mm, 2.6-μm particles, Kinetex PhenylHexyl column, supplied by Phenomenex (Alcobendas, Spain). Xbridge C18 and Kinetex PhenylHexyl columns were applied to produce the separations of the first case study whereas the Cortecs C18 was applied for the second case study. Two different chromatographic systems were used. For case study 1, a Waters Alliance 2695 system, provided with a 2696 PDA detector, was used, running under Empower® software. UV signals were registered in the range 210–400 nm, with a resolution of 1.2 nm. For the second case study, a Waters Acquity H-Class system, provided with an Acquity PDA detector and a Xevo TQD triple quadrupole spectrometer, was used. UV signals were registered in the range 220–400 nm with a resolution of 1.2 nm, and MS signals were registered in full scan mode in the range 50–650, using positive polarity by taking the signal of the first quadrupole of the detector. This system operates under the Waters Masslynx® software.

Mobile phases were composed in all cases by ultrapure water and gradient grade acetonitrile acidified to pH 3.0 with formic acid. For case study 1 runs, a flowrate of 1.0 mL/min was applied whereas for the second case study, a 0.2 mL/min flowrate was used.

### Raw data processing and calculations

All data treatment and calculations were developed by software programs developed by one of the authors using Dephi 10.4.1, programming language (Embarcadero, Austin, TX, USA). These programs include routines to run MCR-ALS, differential evolution, and Pareto optimality in addition to common chromatographic data treatment and graphical outputs.

In all cases, raw data files obtained in the experiments were directly imported and processed to the above-mentioned programs with no other information or metadata about the sample’s composition. Signals produced by the Empower software were exported as text files and processed directly. Raw data files produced by the MassLynx software were imported by means of the facilities provided by the Mnova V.14 suite (Mestrelab Research, Santiago, Spain). Data sets used in case studies are publicly available in the repository of the University of Santiago de Compostela under the ChromChem research Group materials storage [35].

## Results and discussion

The proposed approach will be demonstrated with the help of two case studies that consider separations using DAD-UV and MS detectors.

### Case study A

The case study A considers a mixture of several phenolic compounds, some of them having quite similar UV spectrum to make the sample a challenging case. This sample was evaluated using two chromatographic columns: a fully porous Waters Xbridge C18 and a core-shell Phenomenex Phenyl hexyl. The organic modifier was acetonitrile and mobile phases were acidified to pH 3.0 with formic acid. In addition to gradient time and modifier percentage, the temperature was considered as a continuous variable. The experimental design for such experiment requires 8 runs plus one validation experiment and has been reproduced in Table S1 (see ESM). Maxplot chromatograms corresponding to these 9 runs have been reproduced into Figure S4 (see ESM). Because no information is available about the sample components, the maxplot representation is used to show the run chromatograms and to perform the peak picking process in order to avoid false negatives that could take place by an inappropriate selection of the wavelength channel. This representation mode has however a certain tendency to produce artifact peaks as can be seen in the first part of runs (see maxplots for column A in ESM Figure S4). These signals may appear significant and sometimes higher than analyte peaks but can be easily filtered out during the component’s discovery process because they exhibit a spectral signal highly correlated (positively or negatively) to the maxplot chromatogram background signal (see ESM Figure S5). Excluding the false peaks appearing before the broad signal at the beginning of the chromatograms in column A, no more than 7–8 peaks are evident in most runs, although in some of them, some shoulders and unresolved peaks clearly indicate that the sample would contain more than 8 components.

When these 9 runs were submitted to the component’s discovery process, the results produced are those shown in Table S2 (see ESM). In this table, the following information about each discovered component (a total of 13 initially) is given: (a) the estimated spectrum of the component as proposed by the MCR-ALS algorithm; (b) the goodness of fit for the retention model calculated for this component in all runs; (c) the retention times of each component as assigned in each chromatogram; and (d) the alerts issued for the component in each chromatogram. It is important to appreciate that only the calibration runs (1–8) are used to build the retention models shown in the third column of Table S2 (see ESM). The validation run (9) never enters in the model’s calculation and, thus, serves to assess the predictive ability of the model. In these model plots, the datum for the currently selected run is indicated by means of the filled symbol and by the top-right caption of the graph. The evaluation process of the produced results requires the use of all this information to be reliable. To illustrate this assertion, some situations in ESM Table S2 would be discussed in more detail.

1. False positive components

**Fig. 2:**
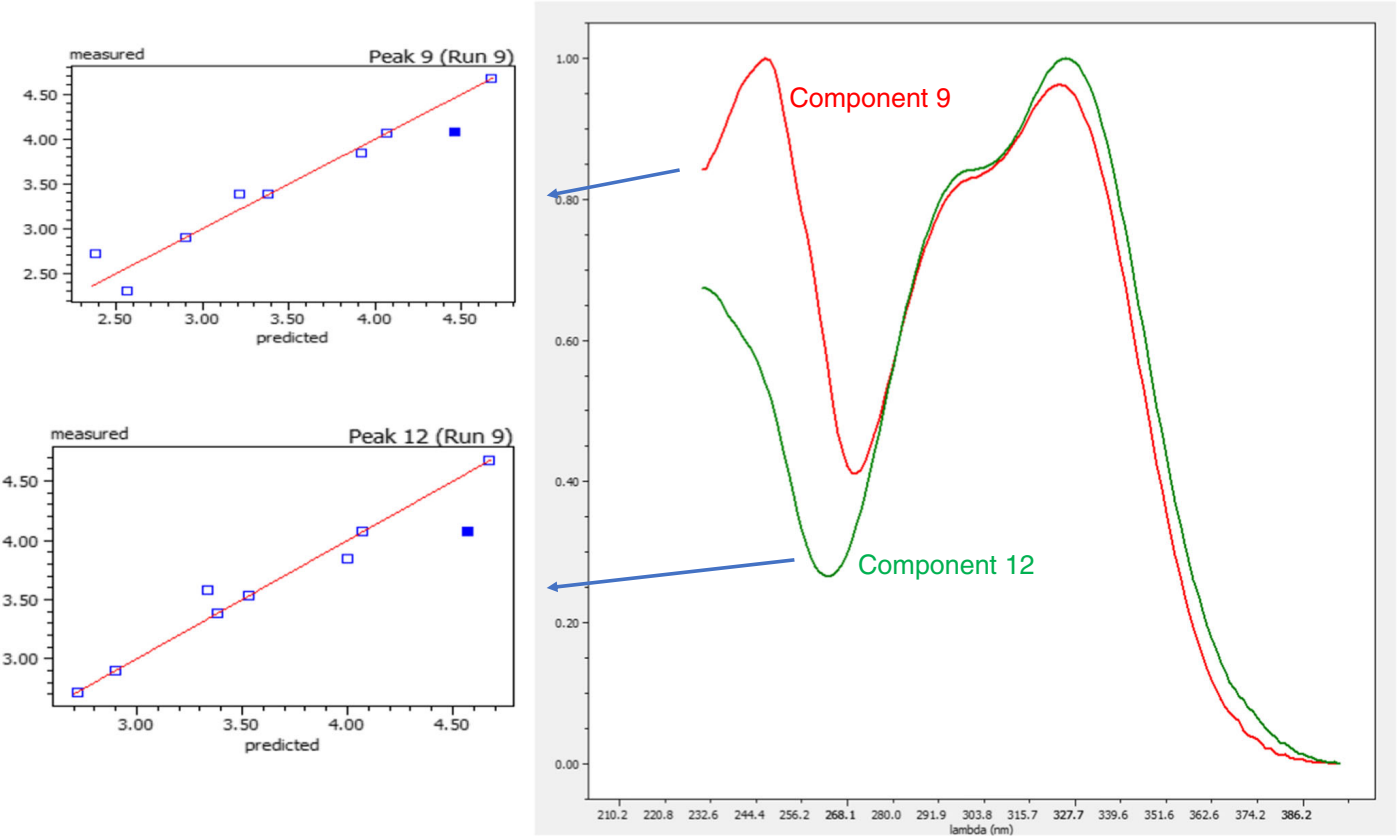
Comparison of spectra for components 9 and 12 (see ESM Table S2 ) in case study A and the corresponding goodness of fit plots with the data for validation run (9) highlighted

In most computer applications implementing the MCR-ALS algorithm, the number of extracted components from the data set is decided by the user by first defining a maximum number of extracted components and then assessing the appearance of the component spectra and profiles. In our case, to provide an automatic operation mode, the following approach was adopted: (1) limit the number of components extracted in each peak to a maximum of six and (2) filtering the extracted components by using an estimation of purity (in UV signals, using the purity value provided by the Simplisma algorithm [24, 25]) and noise (MS signals, calculating the number of signals greater than 0.1% of the base peak in the ESI normalized spectrum) in component’s spectra. This filtering process logically cannot be very stringent to avoid that any true component in the sample should go unnoticed (false negatives). The obvious consequence is that, depending on the mixture complexity, false positive components may appear in the final component’s set that would need to be filtered by inspection at the end of the process by the user. According to the algorithm processing described in the “Theory” section, false positives may result most probably from partially deformed spectra, resulting from component’s extraction processes, which do not pass the correlation limits imposed on component’s spectrum filtering and assignation stages. For example, in ESM Table S2, and Fig. 2 which extracts the information corresponding to the peaks involved, we can see that spectra for components 9 and 12 are very similar so possibly may correspond to the same component. In addition, we see that assigned retention times for both components are also very similar as well as the goodness of fit plots. Thus, the conclusion is that one of these components is redundant and can be removed from the model. Something similar occurs with components 5 and 11 as shown in ESM Figure S6, which exhibit very similar spectra and identical assigned retention times with excellent goodness of fit plots, indicating that both extracted components correspond to the same mixture component. In all cases, the point corresponding to validation run in goodness of fit plot has been highlighted.

2. Unassigned components

**Fig. 3:**
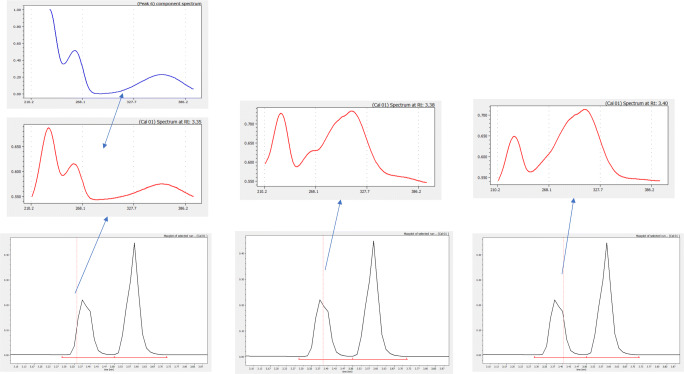
Assessment of unassigned components (blue spectrum corresponds to the lost extracted component. Red spectra correspond—from left to right—to the actual spectra on the left side, apex, and right side of the peak)

Sometimes, the assignment algorithm cannot assign with sufficient accuracy the position of an extracted component because the correlation values between the component spectrum and those of the extracted components in a given chromatogram do not pass the imposed limit (*r* ≥ 0.95 for UV signals). In such cases, the retention time for that component in the chromatogram remains unassigned and the corresponding alert flag issued. In most cases, this is the result of strong and complex overlapping taking place in several runs. In Table S2 (see ESM), we can see two examples of this situation for component 6 and, especially, for component 8. With component 6, the algorithm failed to assign this component in run 1. We see, on the other hand, that retention model (for assigned runs) appears quite good, even for the validation run, and that component spectrum has no other highly similar spectra in the component’s set. The process in these cases consists in locating the component in the already assigned runs and inspects which other components appear overlapped with it. Figure 3 shows the location of the lost sixth component in run 1 following this process. Here, we see that two components are eluted strongly overlapped in the peak at 3.37 min. On the left side of this peak, we can find the spectrum of component 6 (3.35 min). On the right side, we find the other component (3.40 min, which in fact corresponds to component 13 in ESM Table S2). Moreover, in the maximum of the peak, we find a spectrum which is identical to component 1 in ESM Table S2 which has been assigned retention times practically identical to component 13. This clearly suggests that component 1 in ESM Table S2 is not a real sample component but a sum of the totally overlapped components 6 and 13 that was surprisingly assigned with apparent accuracy only in run 1 where component 6 could not be assigned.

**Fig. 4:**
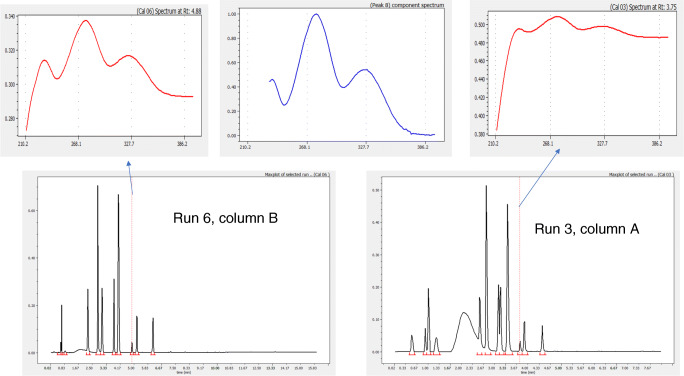
Assessment of unassigned components due to background interference in chromatograms of different columns. Spectra in red correspond to actual spectra on the apex of the selected peak in each chromatogram. Spectrum in blue corresponds to the extracted pure component

The case of component 8 is more complicated. The component could not be assigned in any of the runs corresponding to column A and, on the contrary, was assigned without any apparent difficulty when using column B. Exploring the chromatograms corresponding to column B (see Fig. 4 which compares two runs, one corresponding to each column), we can appreciate that this component eluted as a pure peak in both column B and column A runs, although actual peak spectrum in the runs corresponding to column A appears highly distorted by baseline interference which prevents an accurate assignment of the component in column A runs.

3. Inconsistent retention data

The above discussion with component number 8 is a typical situation of inconsistent retention data, where a component appears easily assignable in one column but not in the other. The goodness of fit plot indicated that retention data measured in the second column fits perfectly whereas for the other column, the lack of satisfactory assignments is evident. Of course, if we tested only one column, this problem would not have appeared. The least information about the mixture composition we have, the more advisable to test several columns although some influence of the column into the UV spectra could require additional user intervention.

4. Alerts on apparently well-fitted data

**Fig. 5:**
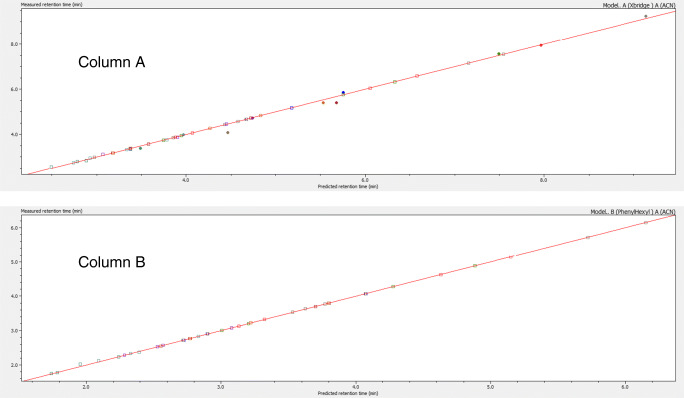
Goodness of fit plots for the overall retention models developed for columns A and B after component’s assignment in case study A

Component 1 diagnostics in Table S2 (see ESM), which we have discussed previously, may represent a good example of situations where a component appears plenty of alert flags although the goodness of fit plot indicates an excellent fit of the retention times measured. These situations are difficult to detect, and frequently, the false positive character of the component is concluded from the study of other assigned components in the sample. As mentioned above, alert flags indicating the potential unrealibility of any assignation are derived from values in the correlation analysis that, being significant in value (e.g., > 0.8), results to be below the acceptance limit (e.g., 0.95 for UV spectra). Thus, it is important to be ready to explore the results if alerts appear, and use all the helping tools provided to develop reliable retention models,

The above-mentioned situations are affected by the sampling frequency in the UV detector signal. The chromatograms for case A were replicated using 1, 2, 5, 10, and 20 points per second sampling frequency. The results indicated that using the minimal sampling frequency, the number of false positives increases, but using sampling frequencies higher than 5 point per second produced identical results. Although this conclusion can be dependent on the sample complexity and the spectrum details of components, it is advisable to use a sampling frequency of at least 5 points per second to facilitate the work of the discovery and assignment algorithms. After these final component filtering processes, we have seen that in fact the sample under study contains 10 components. Components 5 and 9 were discarded by redundancy and component 1 was identified as a false positive. Once the retention times were assigned to components needing revision, the goodness of fit plot for the whole sample components set is shown in Fig. 5 which represents the goodness of fit for the overall retention model for the ten components discovered in columns A and B.

### Case study B

From the above discussion, it is clear that samples containing two or more components with identical UV spectrum can be misassigned sometimes. Although the components may be accurately extracted from one or several runs, the correlation filtering in steps 4 and 5 of the overall assignment processes would filter those spectra highly correlated to define the pure component’s set. This difficulty may pose a serious limitation for the general applicability of the proposed optimization strategy but can be solved by fusioning the spectral information given by the UV signal with that provided by the MS spectrum. Many laboratories doing separation method development have LC systems with DAD and MS detectors in series. LC-ESI-MS instruments provide in most cases very simple mass spectra for components, so the strategy already described needs to be supplemented in (1) step 4 with the MCR-ALS treatment of the total ion chromatogram in the corresponding regions to peak clusters and (2) steps 5 and 6 by modifying the correlation and assignment procedures to include (at least) the base peak in the MS spectrum discovered for each component and preferably the whole MS spectrum (see ESM Figure S3). In this way, components having identical UV spectrum, but different MS spectrum, can be differentiated and, thus, retained in the pure component’s set. Obviously, this condition applies also for components having remarkably similar MS spectra but not identical UV spectra, so the applicability of the proposed strategy becomes significantly enlarged although not universal, because one can find components having the same UV and MS spectra (e.g., enantiomer peaks) in the same run. However, separations involving enantiomers are not the usual objective of the computer-assisted method development in LC, so the proposed strategy can be considered of practical use in most applications being at the same time the first real unified strategy for sample mixtures for known and unknown composition.

To show this extended strategy using UV and MS signals, a really challenging mixture was considered as case study B. This mixture contains 15 phenolic components, some of then having an identical UV spectrum (e.g., caffeic and chlorogenic acids, or 3,4-dihydroxybenzoic and 3,4-dimethoxybenzoic acids), while some others are positional isomers and, thus, have exactly the same base peak in the ESI-MS spectrum (e.g., 2-hydroxycinnamic and 4-hydroxycinnamic acids with MW = 164.16; 4-hydroxy-3,5-dimethoxybenzaldehyde and 3,4-dimethoxybenzoic acid with MW = 182.17; 4-hydroxybenzaldehyde and 3-hydroxybenzaldehyde with MW = 122.12; 2,5-dihydroxybenzoic and 3,4-dihydroxybenzoic acids with MW = 154.12). This means that a strategy based only on UV signals or only in MS signals may have serious difficulties to resolve this mixture satisfactorily, even if several of these peaks are resolved by the column, when the identity of the sample components is not provided to the model and, particularly if do not have the individual standards to verify the retention times of each component. To separate this mixture, a short 50.0 × 3.0-mm Waters Cortecs C18 core-shell column was used. In this case, only the gradient time was considered for the separation using the experimental matrix depicted in Table S3 (see ESM). See the “Experimental” section for elution and data treatment details.

Table S4 (see ESM) shows the result of the component’s discovery process for that case study. Seventeen components are guessed as present in the sample, which in principle is a satisfactory starting point taking into account the sample complexity. In addition, one of the proposed components (peak 3) appears obviously as a background signal so it can be rejected from the set. Peak 4 appears also suspicious because (1) the assigned times are exactly the same as for peak 10; (2) the MS base peak is exactly the same for peaks 10 and 4, and (3) the UV spectrum may correspond to a mixture of spectra for peaks 10 and 8 which are eluted fully overlapped in the third run which was the one where component 4 was unequivocally assigned. Peak 8 (in fact, 4-hydroxybenzoic acid) has an intense UV spectral signal but a very tiny MS signal. On the other hand, it can be seen that several components were not accurately assigned in run 3, and so the user intervention is needed to verify these unsure assignations. The reason resides in the high complexity of run 3 which dumps all peaks in the sample in less than 5 min, thus exhibiting extensive overlapping between components, so the correlation filtering limits imposed in the assignment algorithm are frequently not met, thus triggering the corresponding alerts. In general, test runs are defined to validate the retention model and not to help the discovery process. The idea is to have test runs using elution programs quite different from those used to calibrate the retention model, so at least one slowest and other fastest elutions are programmed. On the contrary, for the discovery process, it is evident that the less overlapping between components, the better. Thus, to reduce the user intervention, it is advisable in these processes to define test runs having intermediate characteristics between those more convenient for testing the retention model and those more useful for the discovery processes. In any case, having the UV and the MS spectral information for components as well as the plot for the partial retention model is quite easy to verify the retention time assignations in all runs (see ESM Figure S7 for a view of these helping tools, which for each run provides visual diagnostics of the component and actual spectra in UV and MS, a goodness of fit plot for the selected component and alert messages, if exists. Moreover, the program does not allow building the retention model if any critical alert has not been addressed), to build dependable retention models. ESM Figure S7 shows also the process to interactively assign a component that was unassigned automatically by the algorithm. In ESM Figure S7(a), we see the starting situation with component unassigned (the red crosshair is situated at the beginning of the chromatogram and the alerts table in the top indicated the failure in component assignation). In part (b) of this figure, the peak was assigned using the trace for the base peak of the component. Now we see that this peak was totally overlapped with another one and partially with a third one so the actual UV spectrum appears quite different to the one extracted for the component. However, the goodness of fit plot is now indicating a good model, thus confirming that the component has been correctly assigned.

**Fig. 6:**
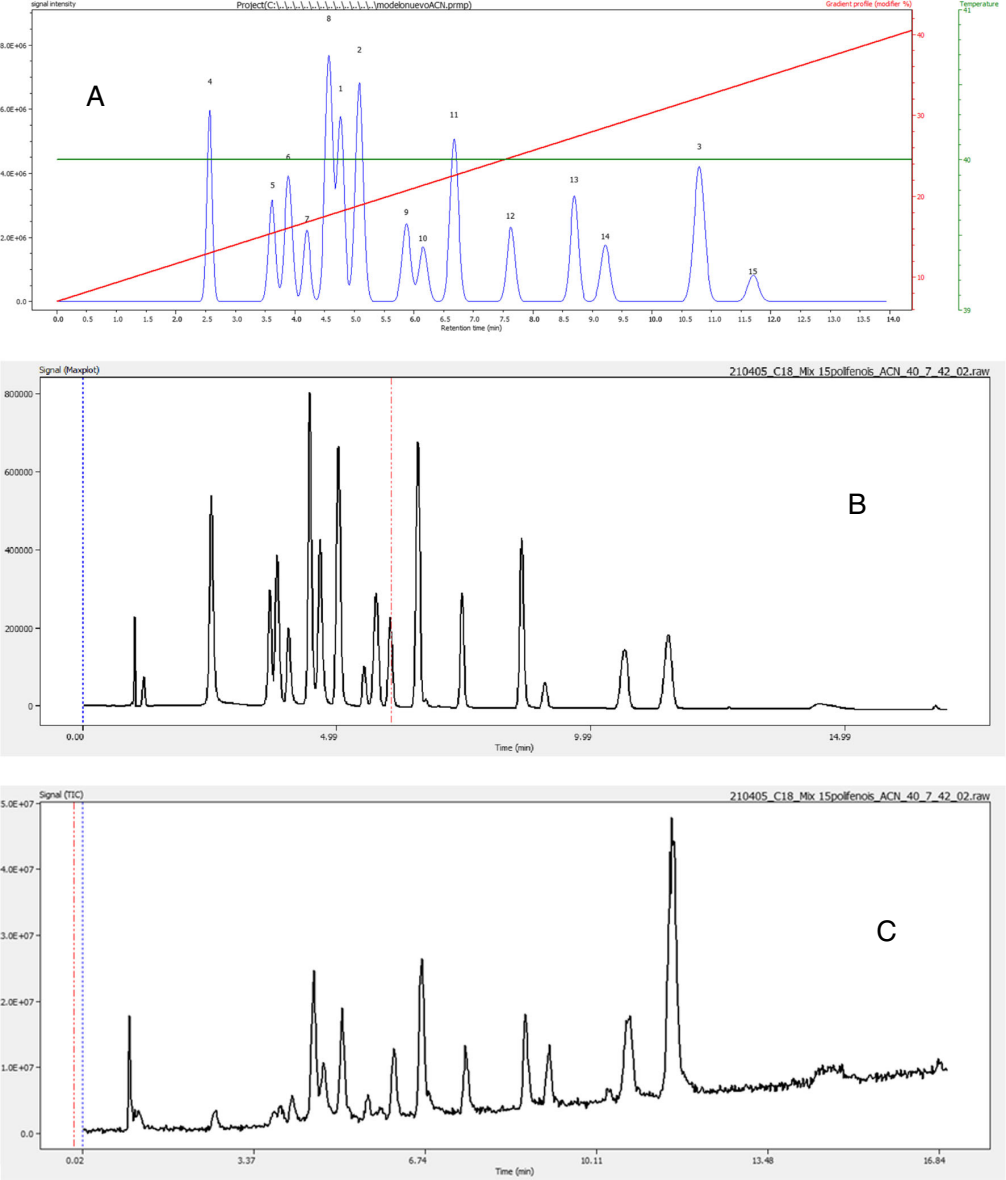
Experimental verification of the optimal separation proposed in case study B. (A) Simulated chromatogram for the optimal separation (7–42% of modifier in 15 min). Peak numbers correspond to the order of discovery for components in the mixture. (B) DAD-UV maxplot of the optimal separation. (C) MS TIC of the optimal separation

Figure S8 (see ESM) shows the goodness of fit of the model built after this verification process for unsure retention times. In this plot, each component is associated with a different color and filled symbols correspond to validation runs. Because the retention model appeared satisfactory, an optimization process using Pareto optimality [32] was launched to establish the optimal elution conditions. Figure 6 compares the proposed optimal elution, showing the simulated chromatogram (A), the UV maxplot (B), and the MS TIC (C). Apparently, all the 15 components of the sample can be resolved but not all of them to baseline confirming the discovery process results about the number of components and the feasibility of the separation using the selected chromatographic column.

## Conclusions

A new and efficient strategy to allow computer-assisted method development in reversed-phase liquid chromatography of unknown mixtures has been developed. For the first time, a unified strategy can be applied to develop separations of mixtures with known and unknown composition. The key elements in this strategy are the use of MCR-ALS to extract the spectral information of the components in the mixture from the raw data files of screening chromatograms registered and, then, use such information to isolate a set of components in the mixture using correlations between these component spectra in the different runs. This allows the accurate evaluation of the retention of such components and, in this way, the calculation of a retention model which is the basis of the simulation processes allowing the optimization of the separation for the components in the mixture. Two case studies of considerable complexity were used to demonstrate the feasibility, efficiency, and limitations of this new approach. Moreover, the same approach can be used if the composition of the mixture is known, because simply the first stages in the component discovery process should be omitted. However, the use of MCR-ALS extracted components instead of the actual spectra in the peak of chromatograms allows improving the accuracy in the detection of components in chromatograms and thus improves also the accuracy in the retention time evaluation also in cases of mixtures of known composition. Consequently, because both procedures, for known and unknown mixtures’ composition, share all elements and algorithms except those devoted specifically to the discovery processes, this new strategy provides the first unified approach in computer-assisted method development for RP-LC.

## Supplementary information

ESM 1 (DOCX 1274 kb)

## Data Availability

Custom code
