# Supplementary material for: A new strategy for the computer-assisted development of reversed-phase liquid chromatography separation methods of unknown sample mixtures
Source: Anal Bioanal Chem. 2021 Aug 18;414(1):587–600. doi: 10.1007/s00216-021-03538-7 (PMC8748381; doi:10.1007/s00216-021-03538-7)

**A new strategy for the computer-assisted development of reversed-phase-liquid-chromatography separation methods of unknown sample mixtures.**

R. Cela^1*^, S. Triñanes^1^, C. Cobas^2^

^1^ Analytical chemistry laboratory. Research Institute for Chemical and Biological Analyses. University of Santiago de Compostela. Spain.

^2^ Mestrelab Research S.L. Santiago de Compostela, Spain.

Supplementary Information

Table S1. Experimental plan for case study A (modifier interval 5-95% in all runs, except (5-63%) in run 9, mobile phase flow rate 1.00 mL/min, other conditions see text). Runs 1-8 were used to calibrate the retention models. Run 9 was used only for validation purposes.

| Run | Column^a^ | Gradient time (min) | Temperature (ºC) |
| --- | --- | --- | --- |
| 1 | A | 7 | 40 |
| 2 | A | 21 | 40 |
| 3 | A | 7 | 55 |
| 4 | A | 21 | 55 |
| 5 | B | 7 | 40 |
| 6 | B | 21 | 40 |
| 7 | B | 7 | 55 |
| 8 | B | 21 | 55 |
| 9 | A | 21 | 44 |

^a^ A = Xbridge C18 column; B = PhenylHexyl column

Figure S1. Schematic view of the MCR-ALS process.


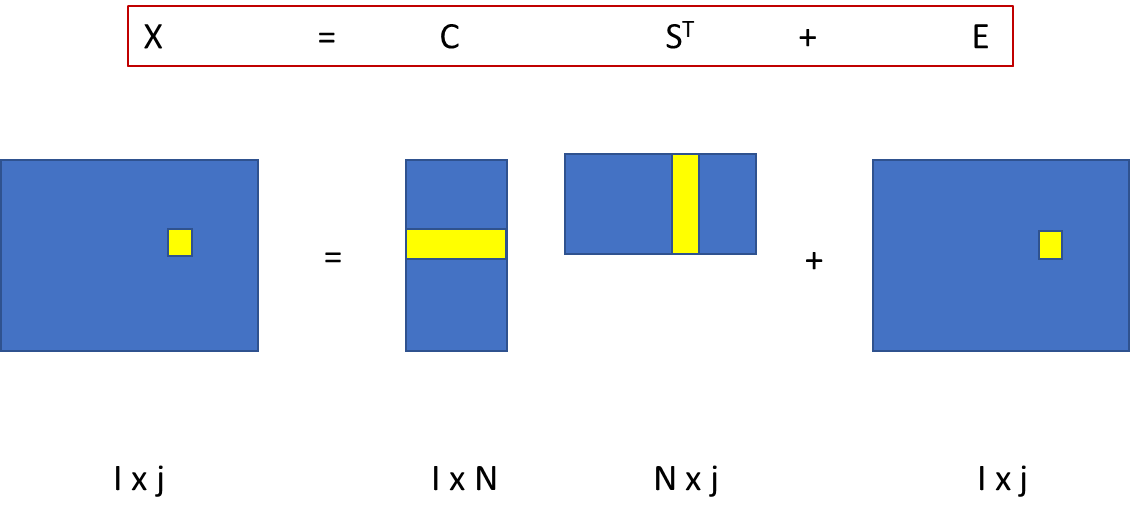


Figure S2. Simplified flow chart of the algorithms adapting the MCR-ALS process to the Step 4 in the proposed strategy.


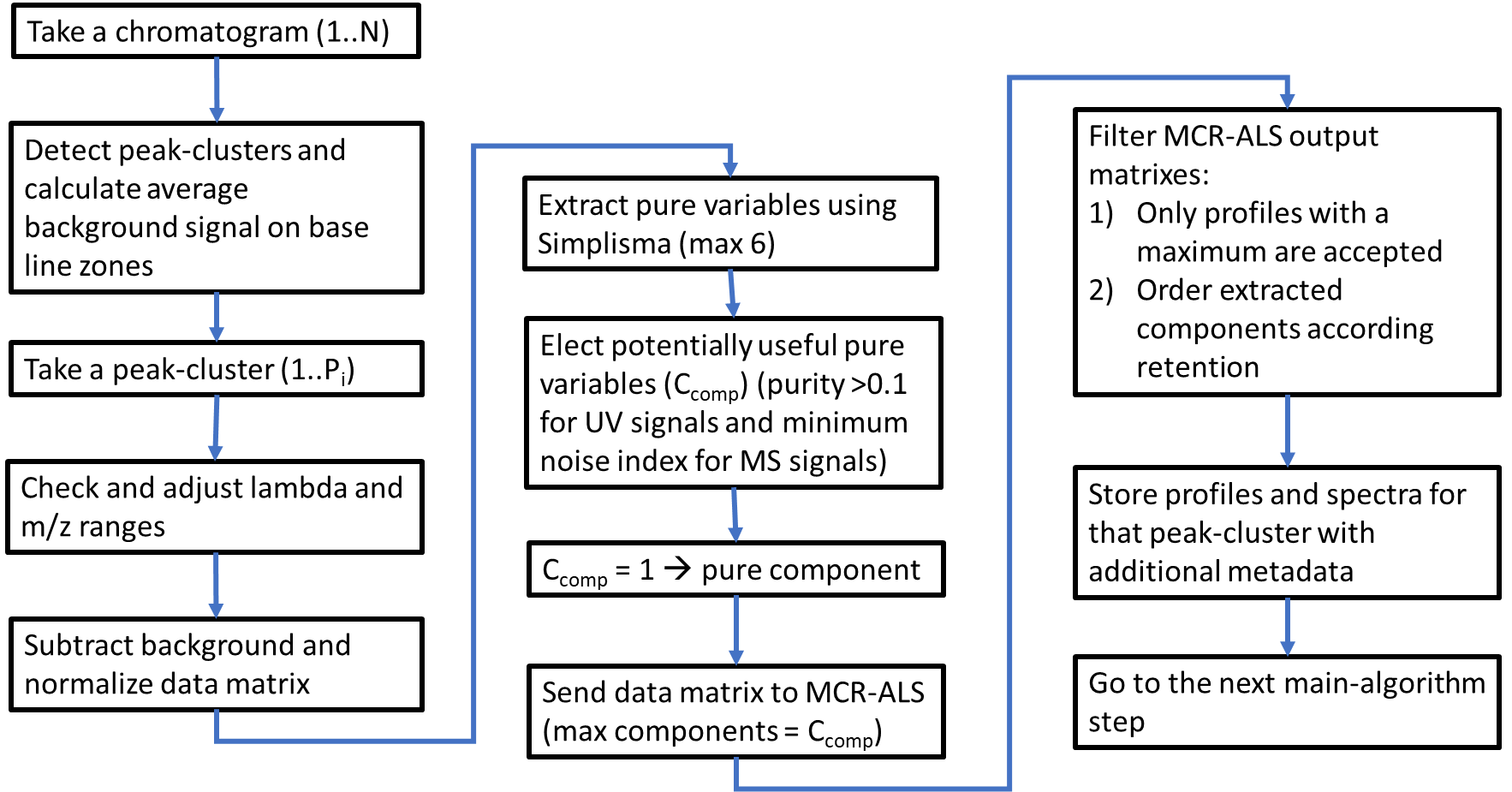


Figure S3. Simplified flow chart of the extraction of the pure components set performed in steps 5 and 6 of the proposed strategy.


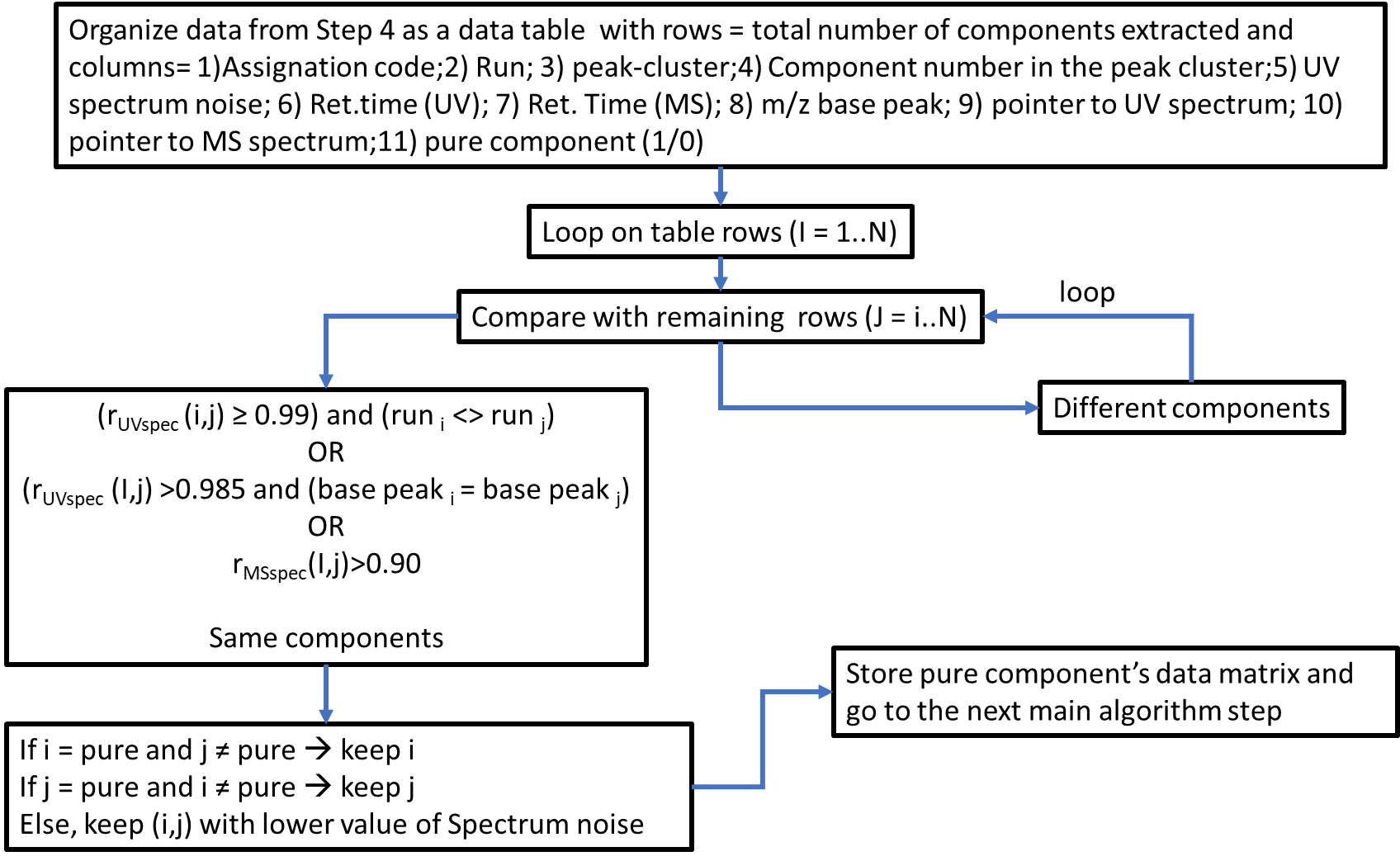


Figure S4. Maxplots corresponding to runs in table S1.


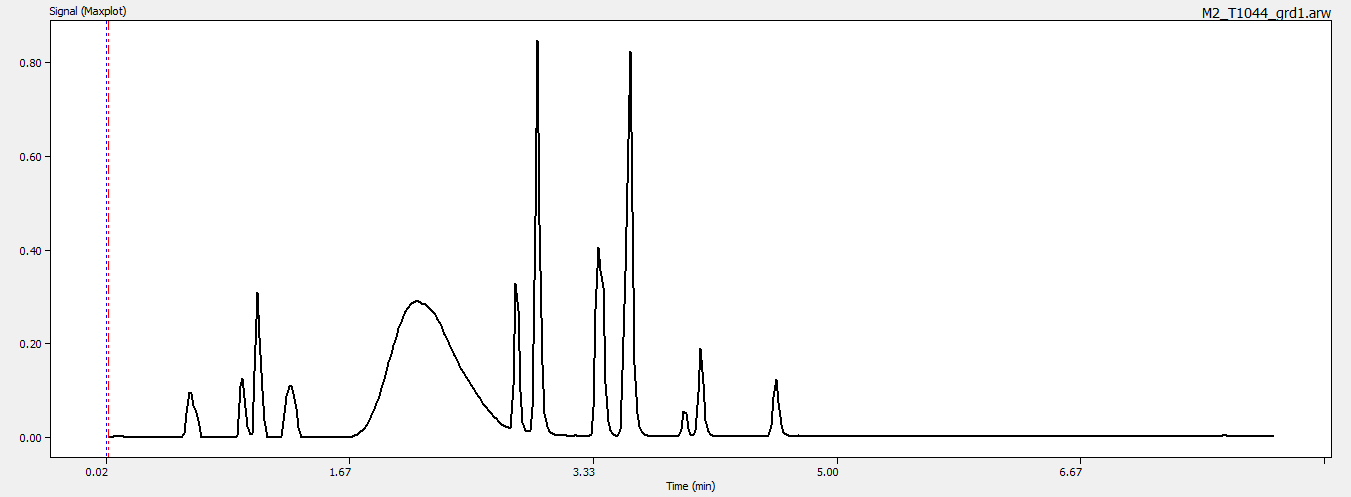


Run 1


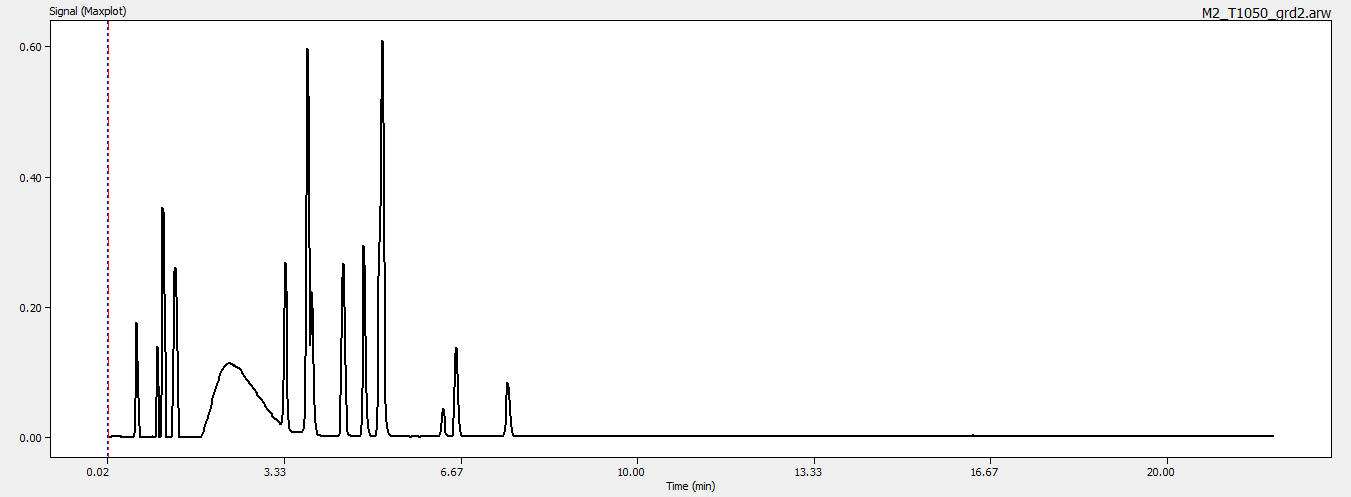


Run 2


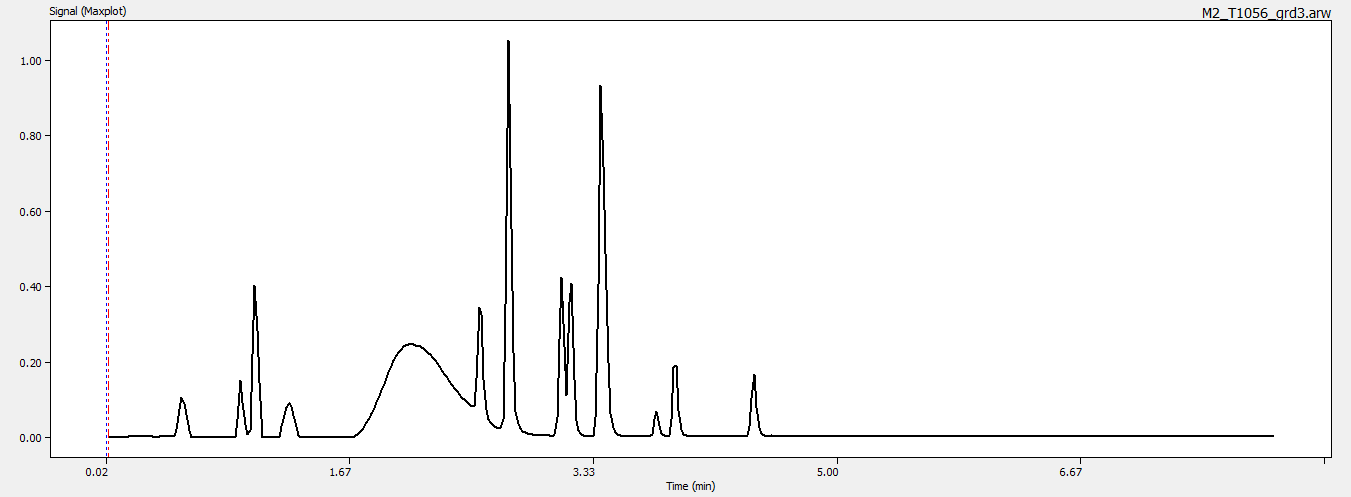


Run 3


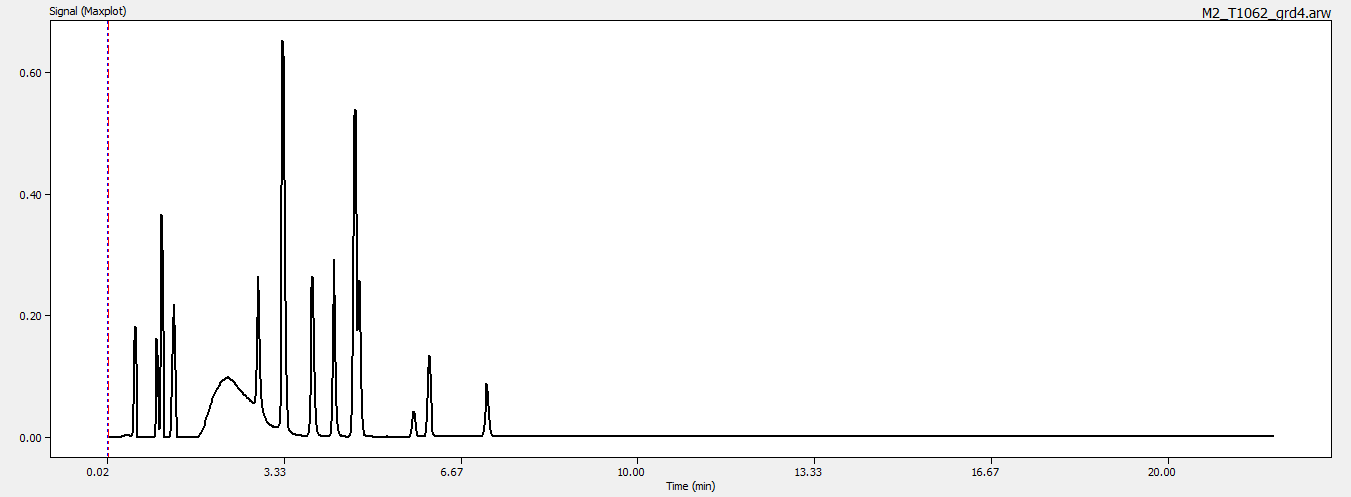


Run 4


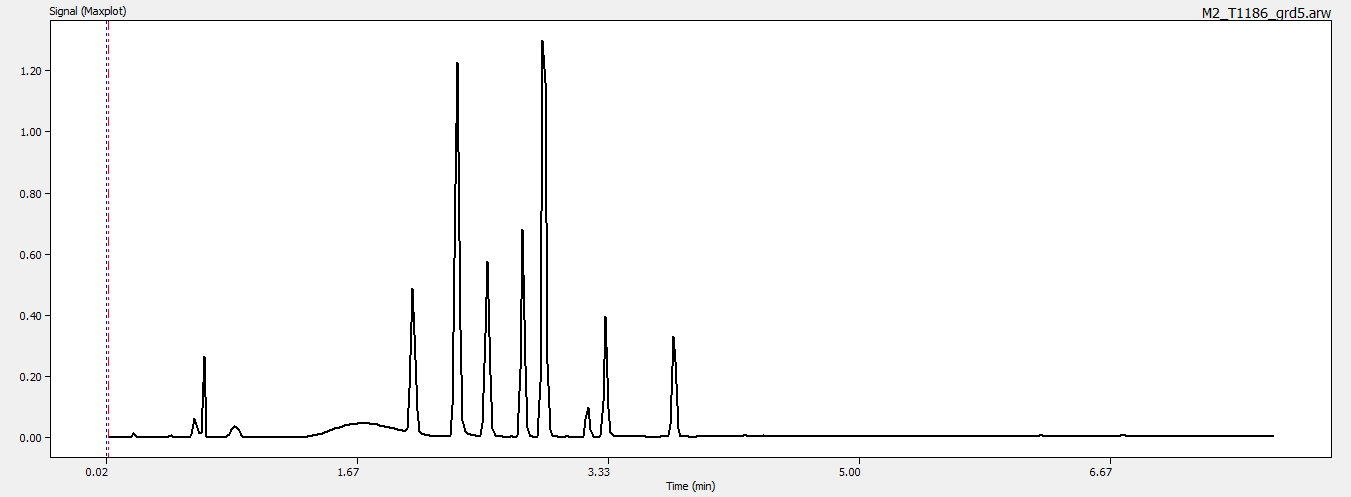


Run 5


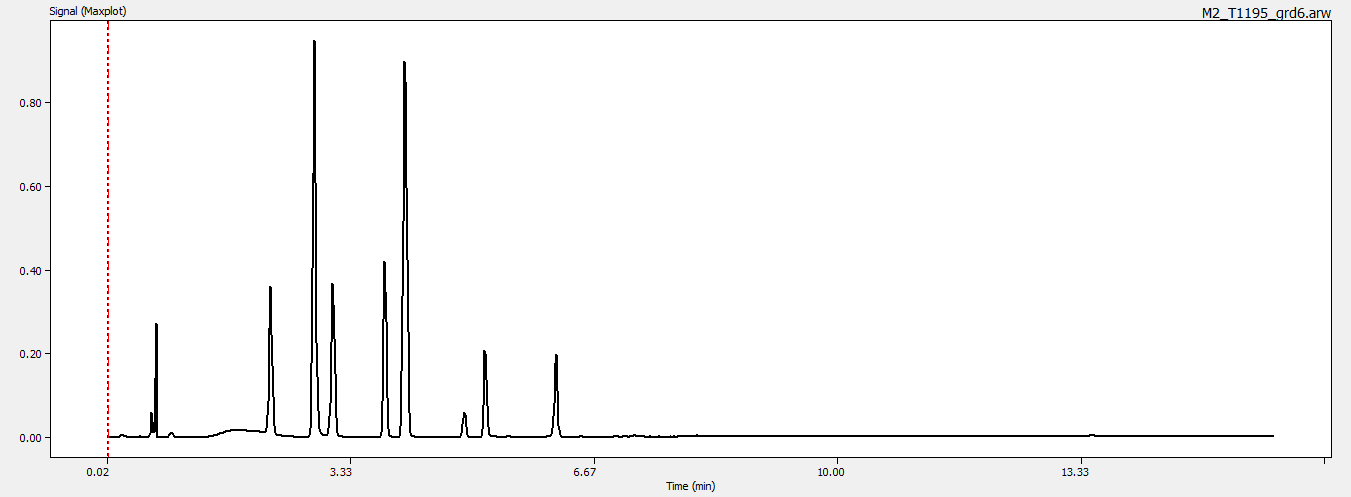


Run 6


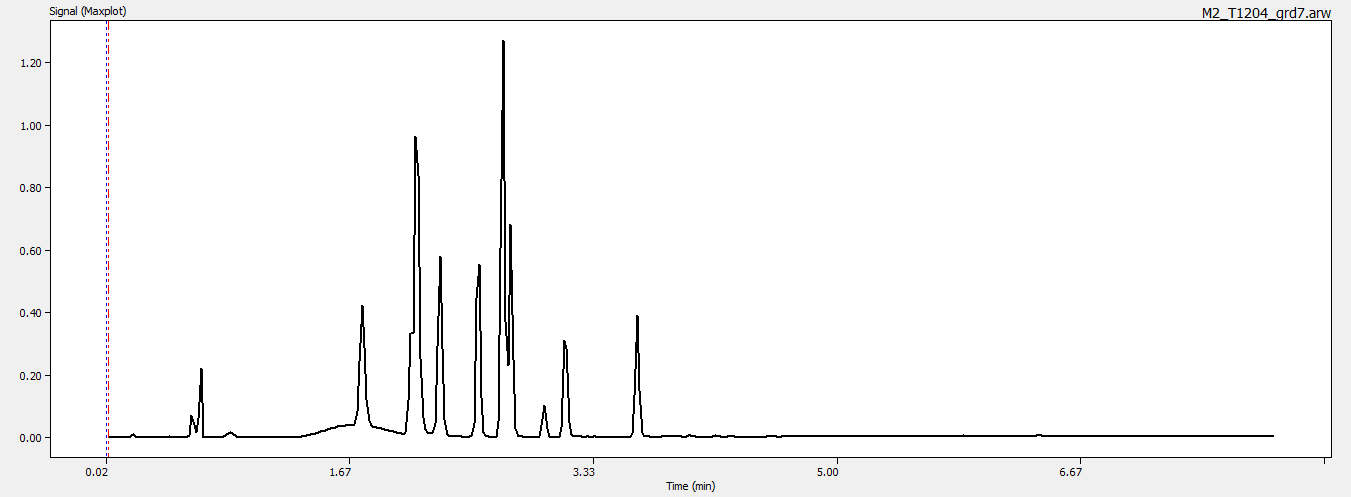


Run 7


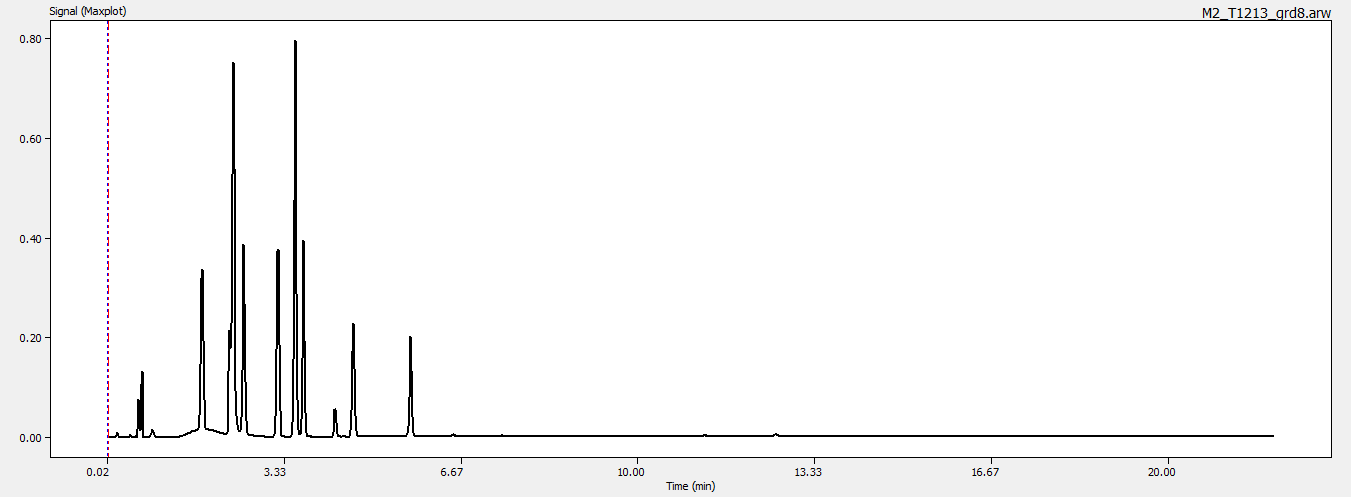


Run 8


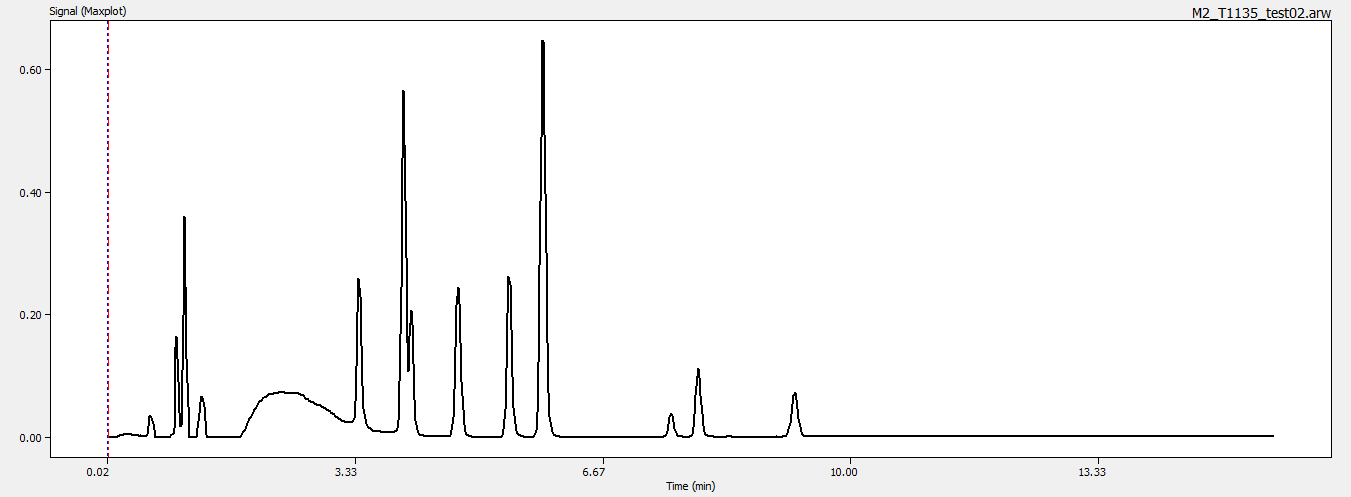


Run 9

Figure S5. False peak spectra due to maxplot artifacts and background spectra in the same run. Absolute correlation coefficients between false peaks spectra and background spectra > 0.98.


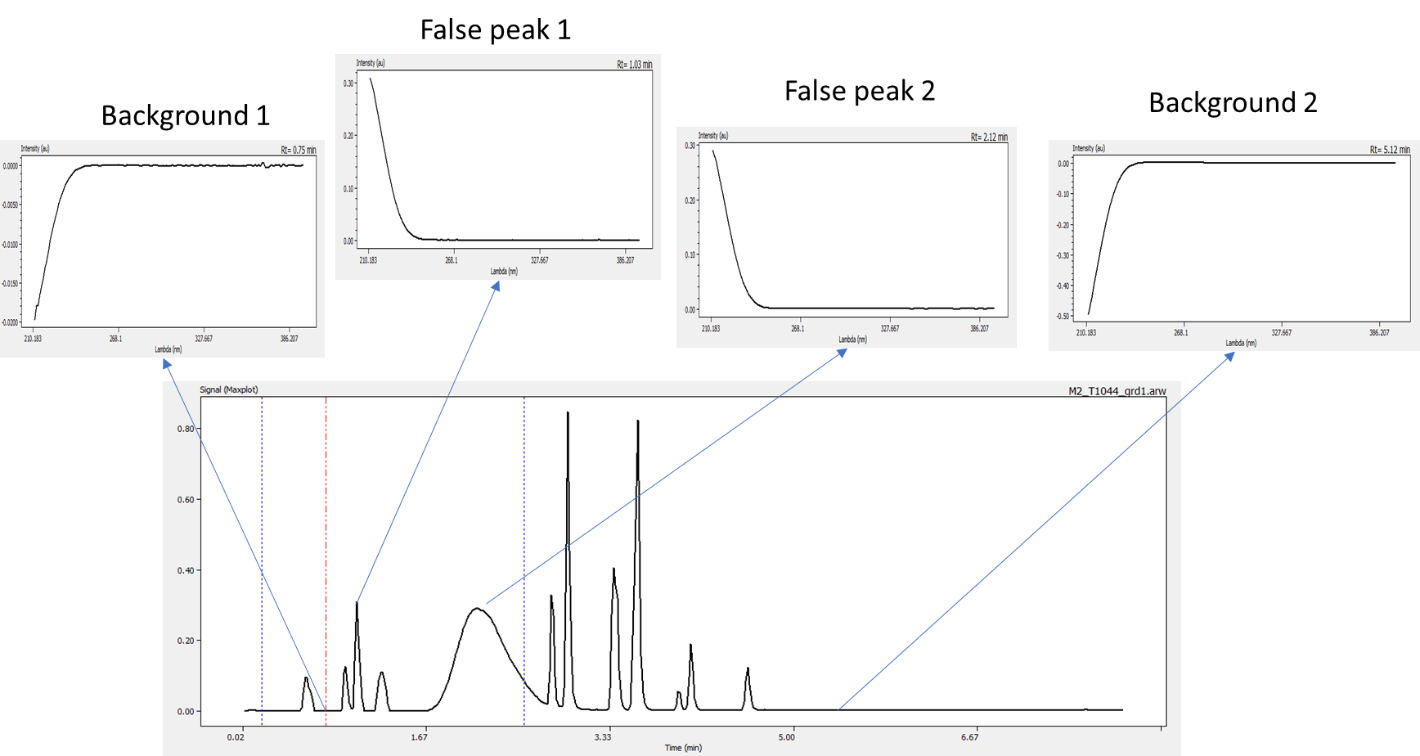


Table S2. Output of the discovery process for case study A

| Component detected | Component spectrum | Goodness of fit diagnostic | Run | | | | | | | | | Information |
| --- | --- | --- | --- | --- | --- | --- | --- | --- | --- | --- | --- | --- |
|  |  |  | 1 | 2 | 3 | 4 | 5 | 6 | 7 | 8 | 9 |  |
| 1 | 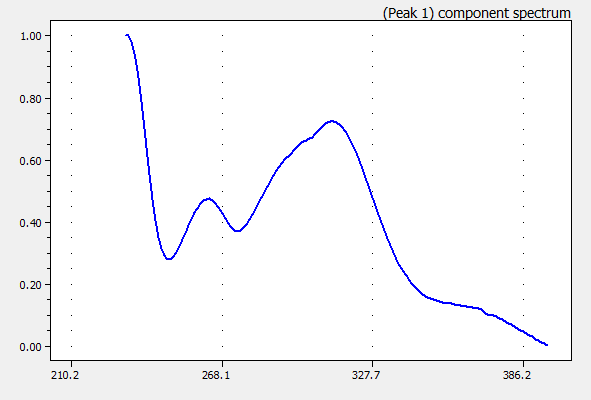 | 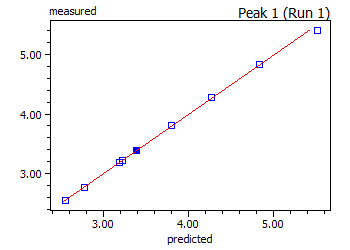 | 3.38 | 4.83 | 3.18 | 4.27 | 2.77 | 3.80 | 2.55 | 3.22 | 5.40 | ret. Time (min) |
|  |  |  |  | a | a | a | a | a | a | a | a | alerts |
| 2 | 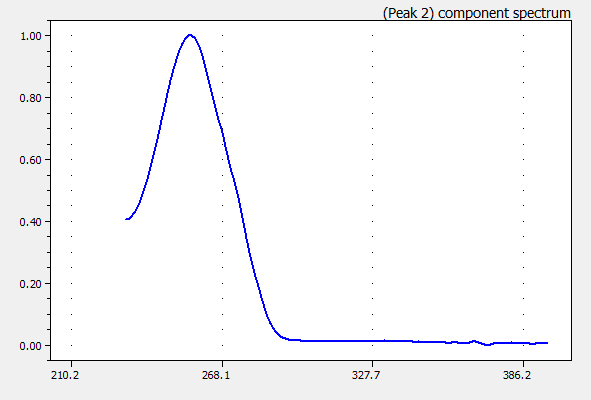 | 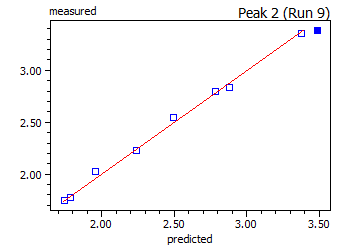 | 2.80 | 3.35 | 2.55 | 2.83 | 2.03 | 2.23 | 1.75 | 1.78 | 3.38 | ret. Time (min) |
|  |  |  |  |  |  |  |  |  |  |  |  | alerts |
| 3 | 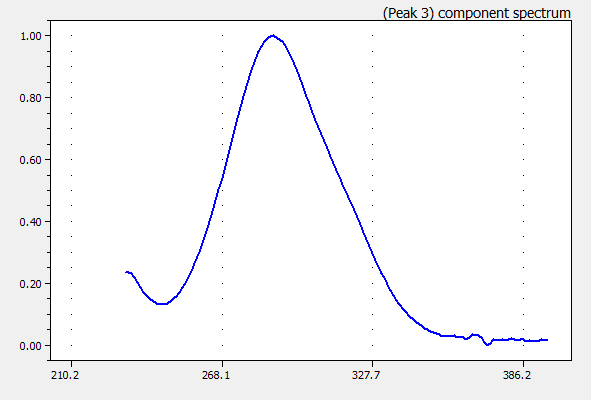 | 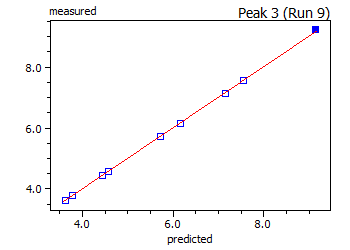 | 4.58 | 755 | 4.43 | 7.15 | 3.77 | 6.15 | 3.63 | 5.72 | 9.25 | ret. Time (min) |
|  |  |  |  |  |  |  |  |  |  |  |  | alerts |
| 4 | 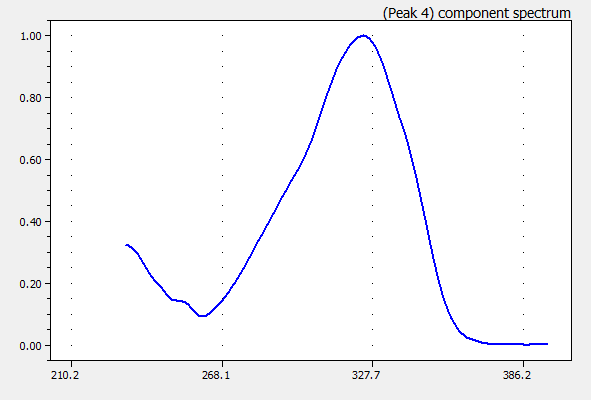 | 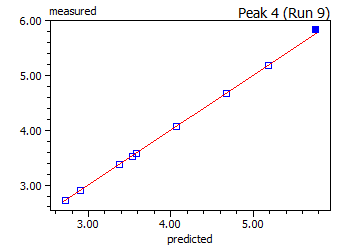 | 3.58 | 5.18 | 3.38 | 4.67 | 2.90 | 4.07 | 2.72 | 3.53 | 5.85 | ret. Time (min) |
|  |  |  |  |  |  |  |  |  |  |  |  | alerts |
| 5 | 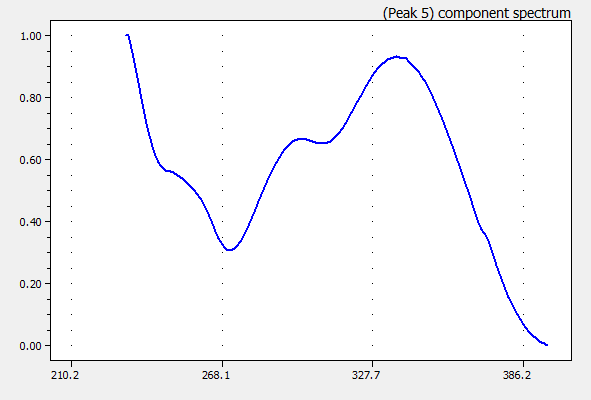 | 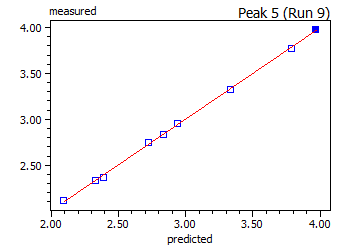 | 2.95 | 3.77 | 2.75 | 3.32 | 2.33 | 2.83 | 2.12 | 2.37 | 3.98 | ret. Time (min) |
|  |  |  |  | a |  |  |  |  |  |  | a | alerts |
| 6 | 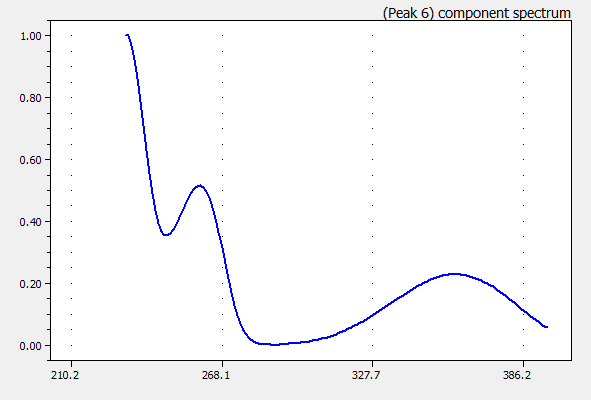 | 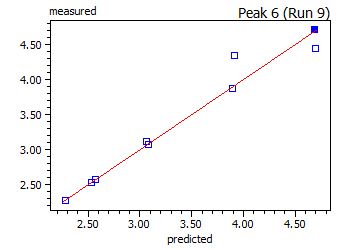 | - | 4.45 | 3.12 | 3.87 | 2.53 | 3.08 | 2.28 | 2.57 | 4.72 | ret. Time (min) |
|  |  |  | b |  |  |  |  |  |  |  |  | alerts |
| 7 | 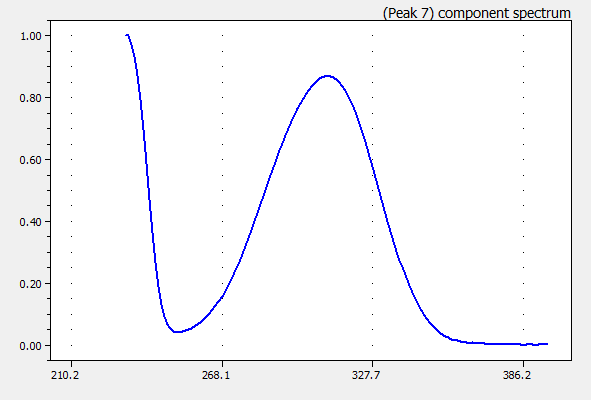 | 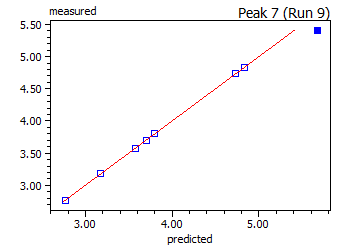 | 3.58 | 4.83 | 3.18 | 4.73 | 2.77 | 3.80 | 2.77 | 3.70 | 5.40 | ret. Time (min) |
|  |  |  | a | a | a |  | a | a |  |  | a | alerts |
| 8 | 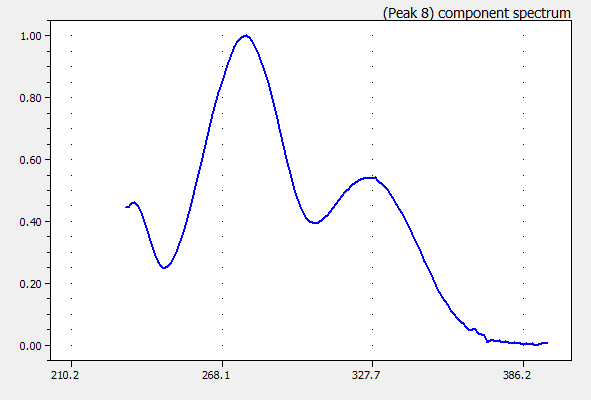 | 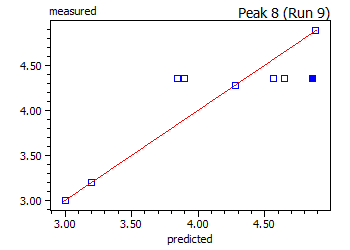 | - | - | - | - | 3.20 | 4.88 | 3.00 | 4.28 | - | ret. Time (min) |
|  |  |  | b | b | b | b |  |  |  |  | b | alerts |
| 9 | 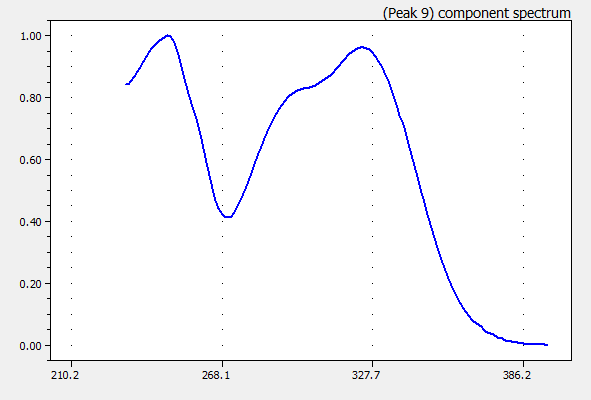 | 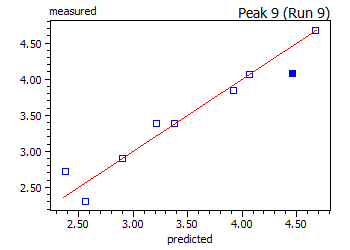 | 3.38 | 3.85 | 3.38 | 4.67 | 2.90 | 4.07 | 2.72 | 2.30 | 4.08 | ret. Time (min) |
|  |  |  | a | a | a | a | a | a | a |  | a | alerts |
| 10 | 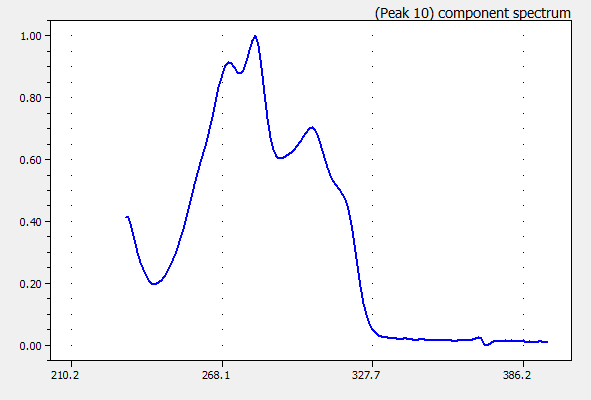 | 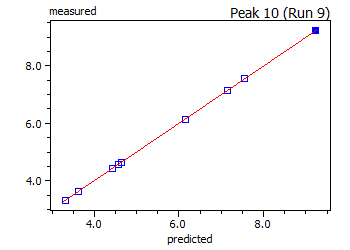 | 4.58 | 7.55 | 4.43 | 7.15 | 3.32 | 6.15 | 3.63 | 4.63 | 9.25 | ret. Time (min) |
|  |  |  | a | a | a | a |  | a | a |  | a | alerts |
| 11 | 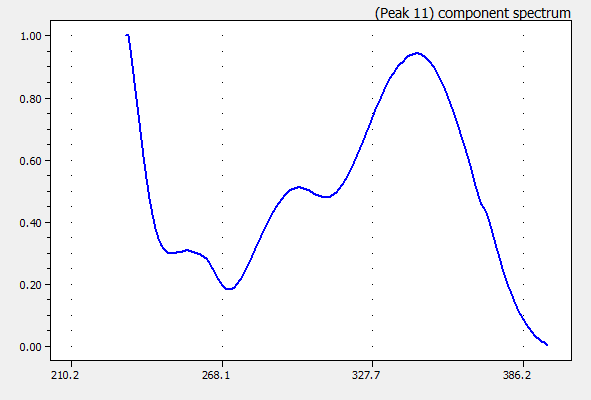 | 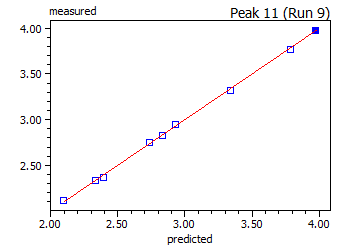 | 2.95 | 3.77 | 2.75 | 3.32 | 2.33 | 2.83 | 2.12 | 2.37 | 3.98 | ret. Time (min) |
|  |  |  | a |  | a | a | a | a | a | a |  | alerts |
| 12 | 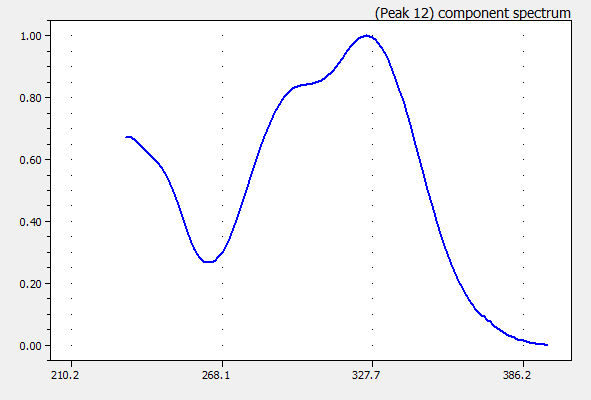 | 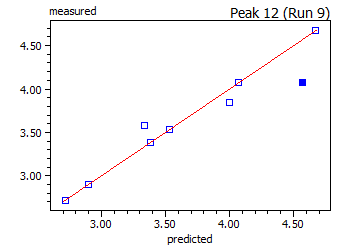 | 3.58 | 3.85 | 3.38 | 4.67 | 2.90 | 4.07 | 2.72 | 3.53 | 4.08 | ret. Time (min) |
|  |  |  | a |  | a | a | a | a | a | a |  | alerts |
| 13 | 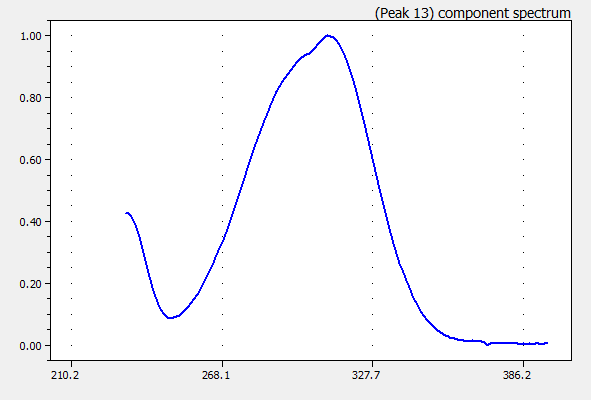 | 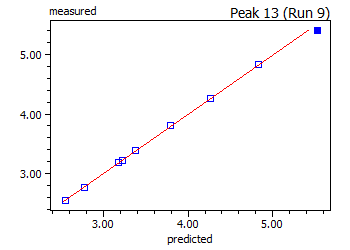 | 3.38 | 4.83 | 3.18 | 4.27 | 2.77 | 3.80 | 2.55 | 3.22 | 5.40 | ret. Time (min) |
|  |  |  | a |  |  |  |  |  |  |  |  | alerts |
|  |  |  |  |  |  |  |  |  |  |  |  | alerts |

1. Assignation may not be reliable, (b) Component could not be assigned

Figure S6. Comparison of spectra for extracted components 5 and 11 in case study A.

| 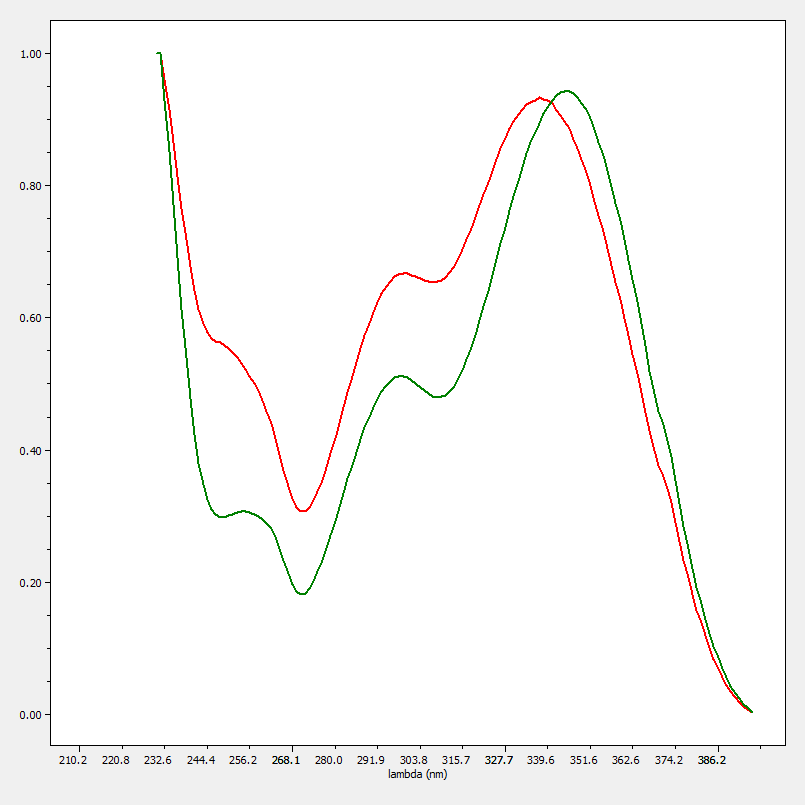  Component 11  Component 5 | 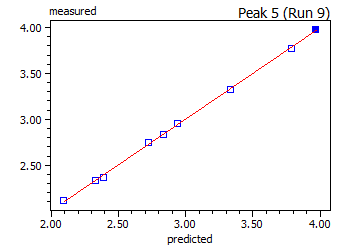 |
| --- | --- |
|  | 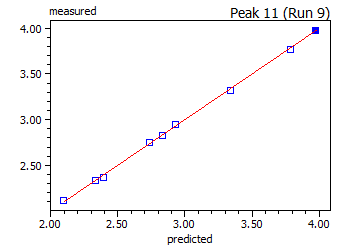 |

Table S3. Experimental plan for case study B (modifier interval 5-95% in all runs, except (5-63%) in run 9, mobile phase flow rate 0.20 mL/min, other conditions see text). Runs 1-2 were used to develop the retention model. Runs 3 and 4 were used only for validation purposes.

| Run | Gradient time | Modifier % start | Modifier % final | temperatura (ºC) | pH |
| --- | --- | --- | --- | --- | --- |
| Cal 01 | 15 | 5 | 95 | 40 | 3 |
| Cal 02 | 45 | 5 | 95 | 40 | 3 |
| Test 01 | 15 | 24 | 95 | 40 | 3 |
| Test 02 | 45 | 5 | 63 | 40 | 3 |

Table S4. Output of the discovery process for case study B

| Component detected | Component UV spectrum | Component MS spectrum | Goodness of fit diagnostic | Run | | | | Information |
| --- | --- | --- | --- | --- | --- | --- | --- | --- |
|  |  |  |  | Cal 01 | Cal 02 | Test 01 | Test 02 |  |
| 1 | 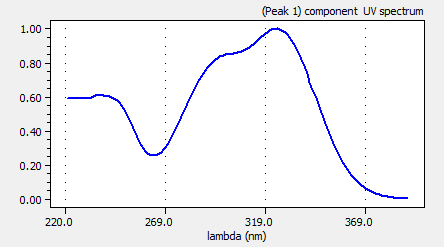 | 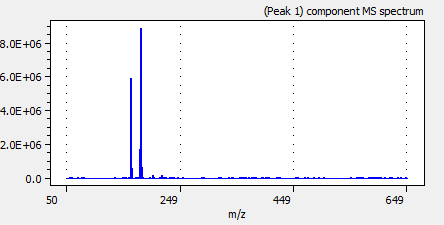 | 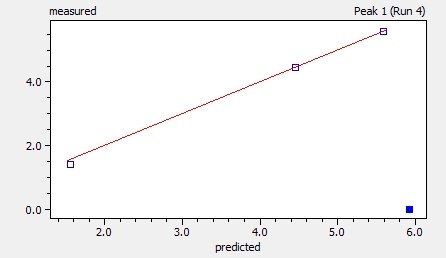 | 4.46 | 5.59 | 1.41 | - | Ret.time (min) |
|  |  |  |  |  |  |  | b | Alerts |
| 2 | 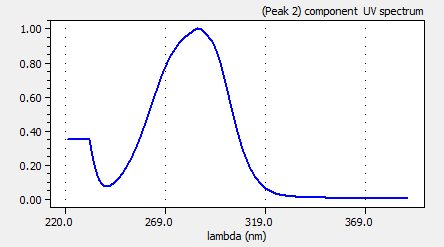 | 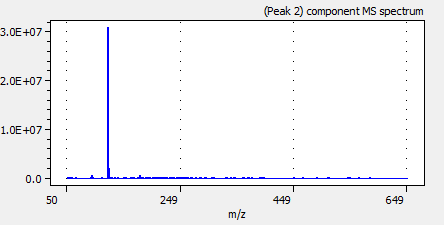 | 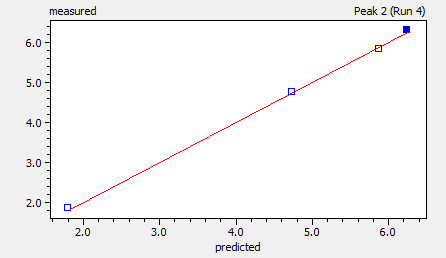 | 4.78 | 5.84 | 1.87 | 6.32 | Ret.time (min) |
|  |  |  |  |  |  |  |  | Alerts |
| 3 | 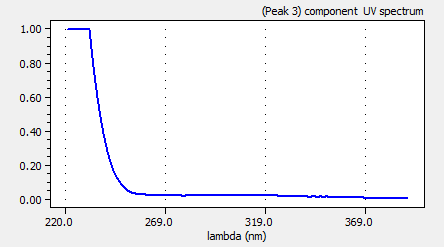 | 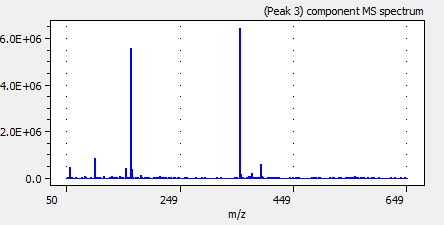 | 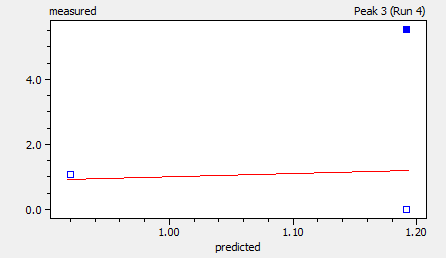 | - | - | 1.09 | 5.53 | Ret.time (min) |
|  |  |  |  | b | b |  | a | Alerts |
| 4 | 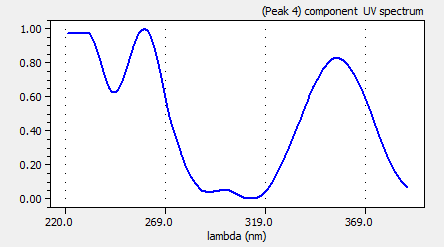 | 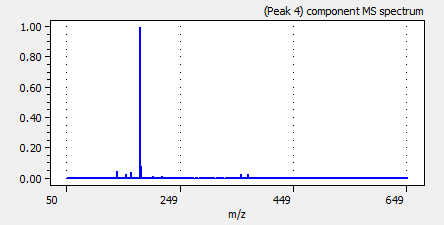 | 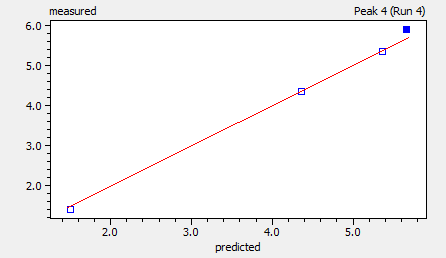 | 4.37 | 5.36 | 1.41 | 5.91 | Ret.time (min) |
|  |  |  |  | a | a |  | a | Alerts |
| 5 | 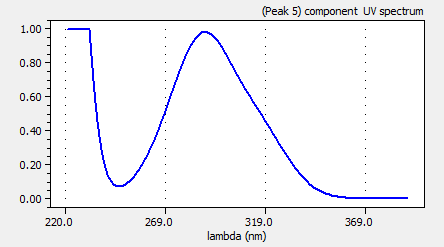 | 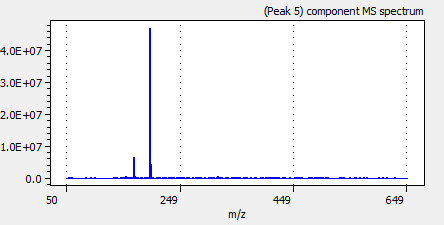 | 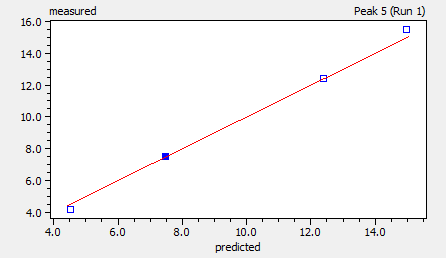 | 7.48 | 12.39 | 4.18 | 15.49 | Ret.time (min) |
|  |  |  |  |  |  |  |  | Alerts |
| 6 | 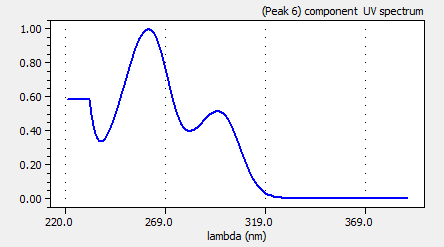 | 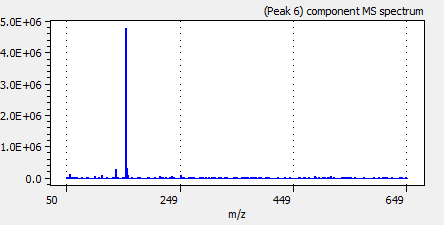 | 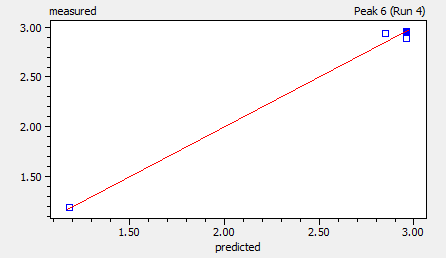 | 2.94 | 2.89 | 1.19 | 2.96 | Ret.time (min) |
|  |  |  |  |  |  |  |  | Alerts |
| 7 | 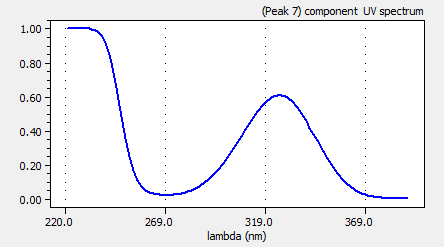 | 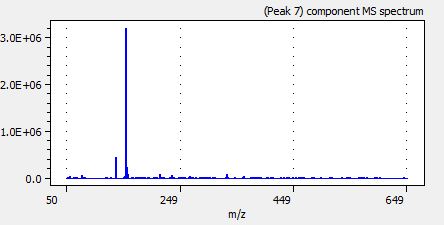 | 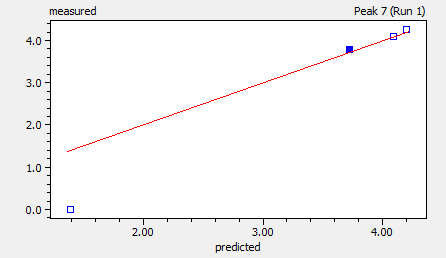 | 3.79 | 4.09 | - | 4.26 | Ret.time (min) |
|  |  |  |  |  |  | b |  | Alerts |
| 8 | 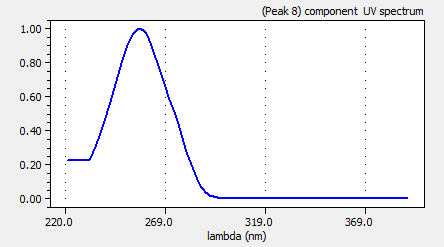 | 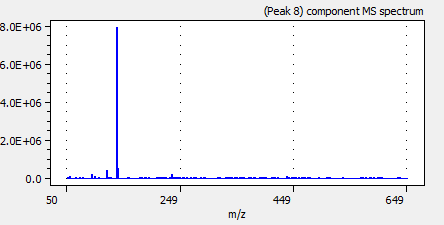 | 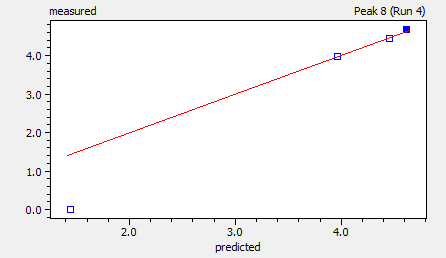 | 3.98 | 4.44 | - | 4.68 | Ret.time (min) |
|  |  |  |  |  |  | b |  | Alerts |
| 9 | 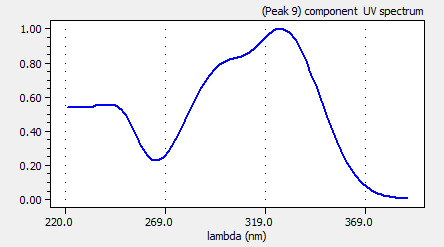 | 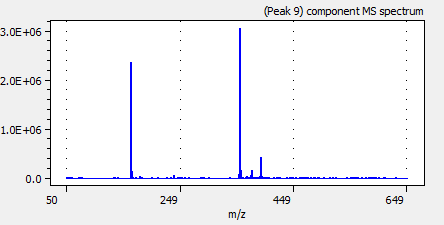 | 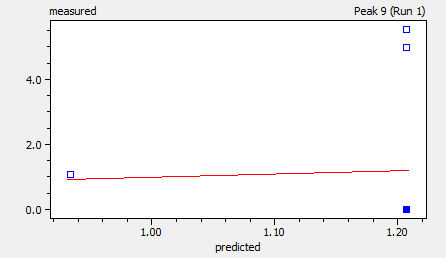 | - | 4.97 | 1.09 | 5.53 | Ret.time (min) |
|  |  |  |  | b |  | a |  | Alerts |
| 10 | 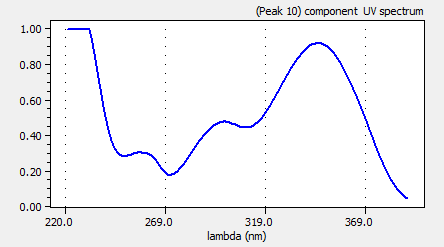 | 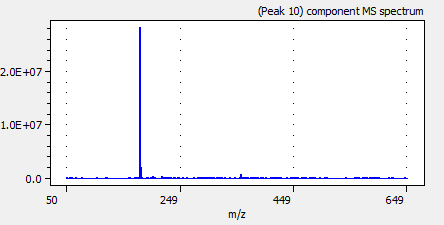 | 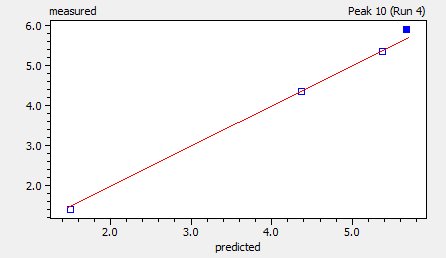 | 4.37 | 5.36 | 1.41 | 5.91 | Ret.time (min) |
|  |  |  |  |  |  | a |  | Alerts |
| 11 | 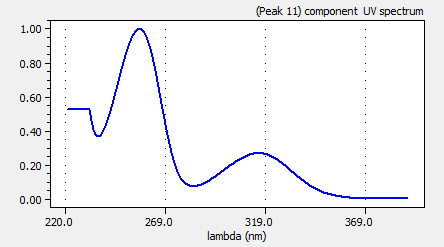 | 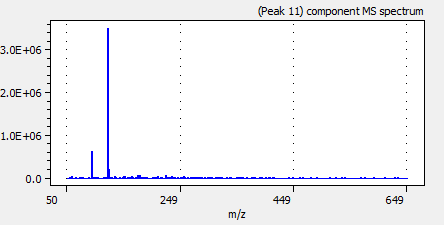 | 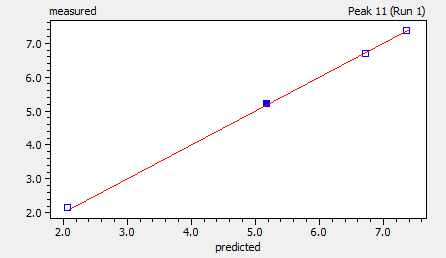 | 5.21 | 6.69 | 2.15 | 7.39 | Ret.time (min) |
|  |  |  |  |  |  |  |  | Alerts |
| 12 | 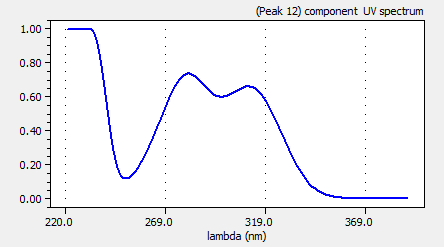 | 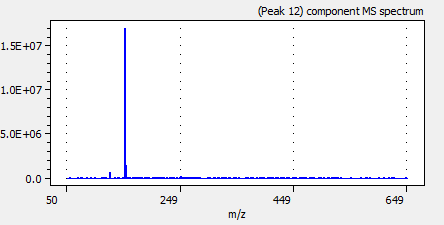 | 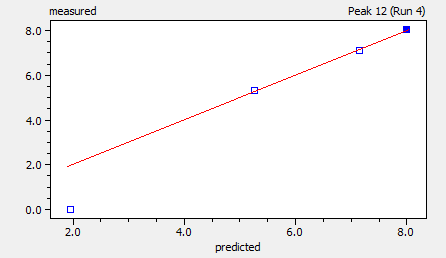 | 5.30 | 7.09 | - | 8.03 | Ret.time (min) |
|  |  |  |  |  |  | b |  | Alerts |
| 13 | 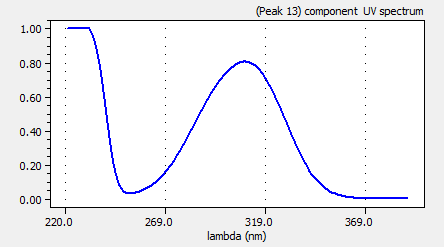 | 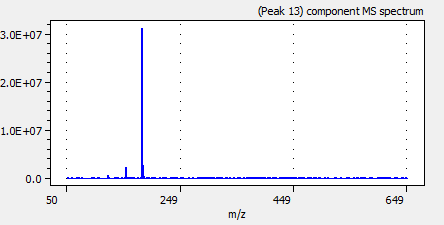 | 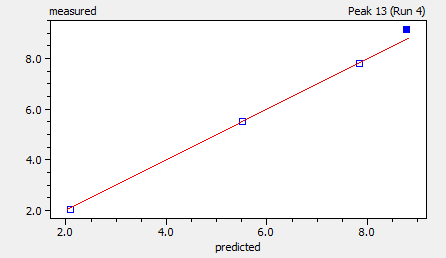 | 5.50 | 7.82 | 2.05 | 9.15 | Ret.time (min) |
|  |  |  |  |  |  |  |  | Alerts |
| 14 | 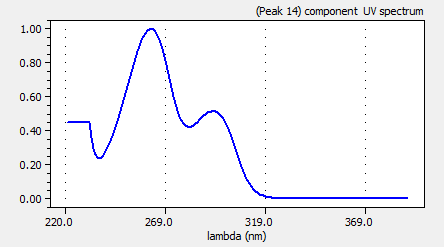 | 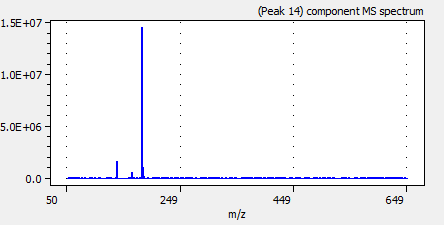 | 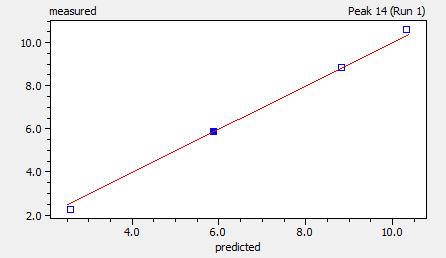 | 5.87 | 8.83 | 2.26 | 10.61 | Ret.time (min) |
|  |  |  |  |  |  |  |  | Alerts |
| 15 | 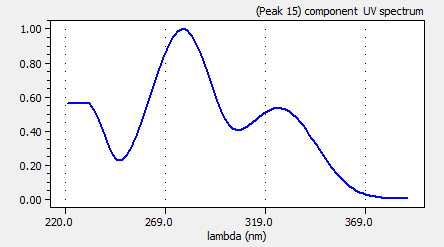 | 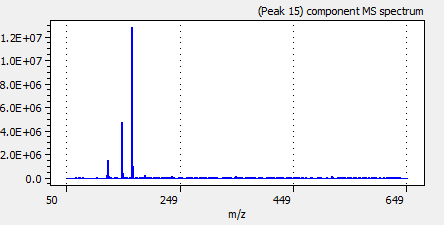 | 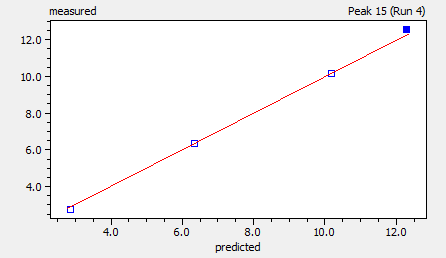 | 6.35 | 10.18 | 2.75 | 12.56 | Ret.time (min) |
|  |  |  |  |  |  |  |  | Alerts |
| 16 | 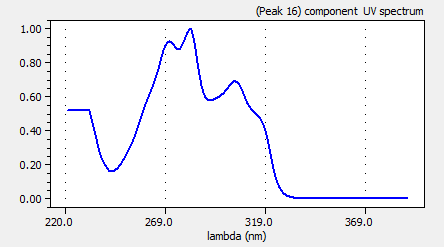 | 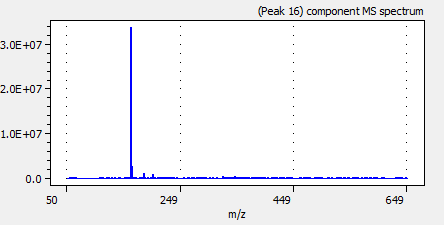 | 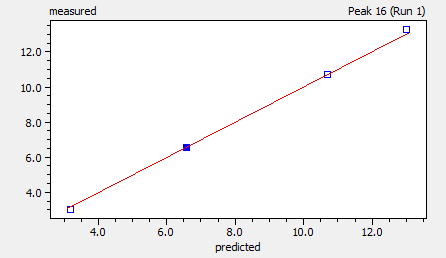 | 6.56 | 10.69 | 3.02 | 13.28 | Ret.time (min) |
|  |  |  |  |  |  |  |  | Alerts |
| 17 | 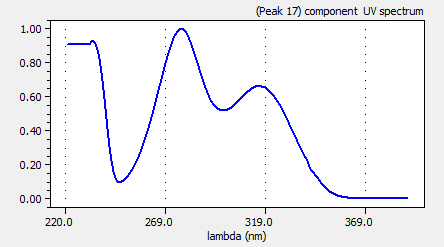 | 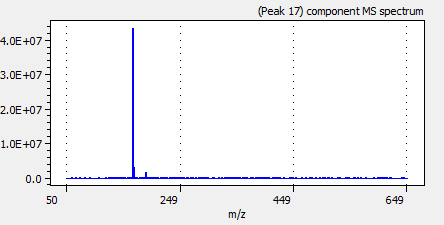 | 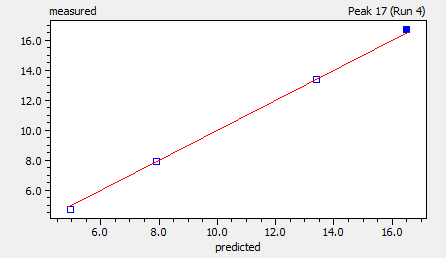 | 7.90 | 13.40 | 4.72 | 16.73 | Ret.time (min) |

1. Assignation may not be reliable, (b) Component could not be assigned

Figure S7. Helping tools in the verification of unsure component’s assignation to peaks in chromatograms. (a) before manual assignation (see alerts and the outlier point in the component partial retention model. (b) after manual assignation of component that was eluted totally overalpped with another component.


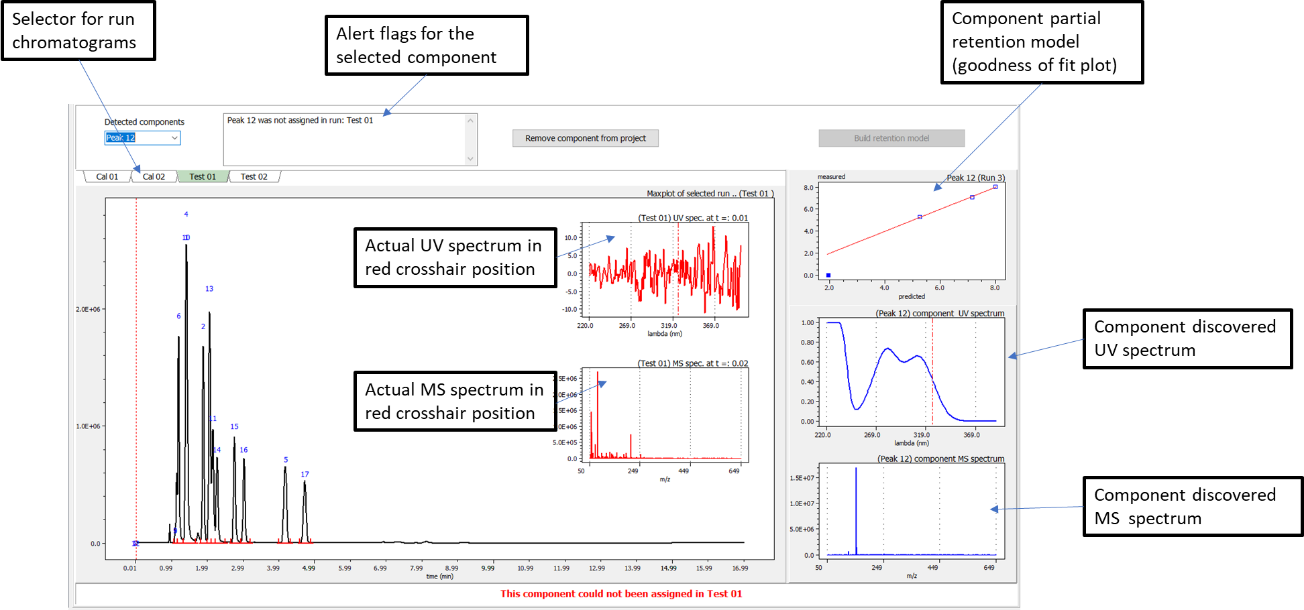


(a)


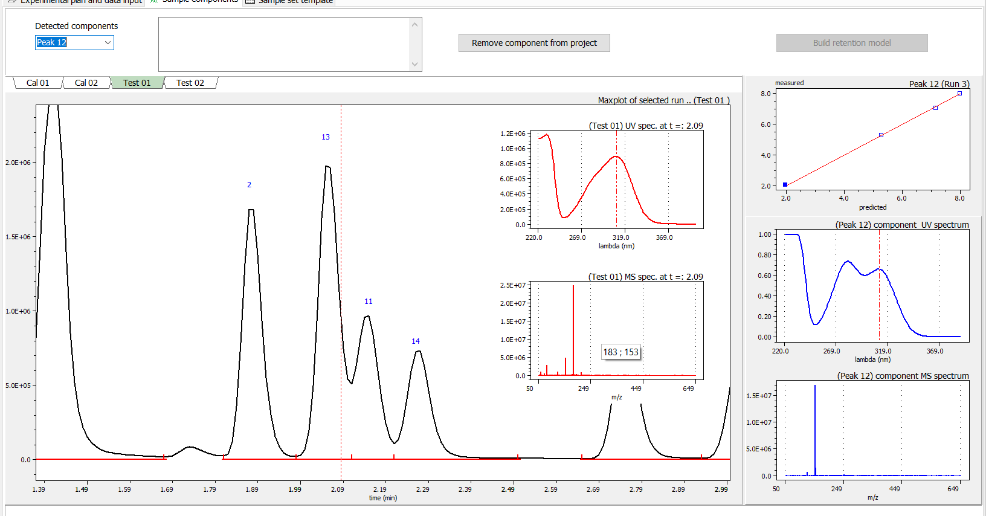


(b)

Figure S8. Goodness of fit plot for the retention model calculated after components assignment in case study B.


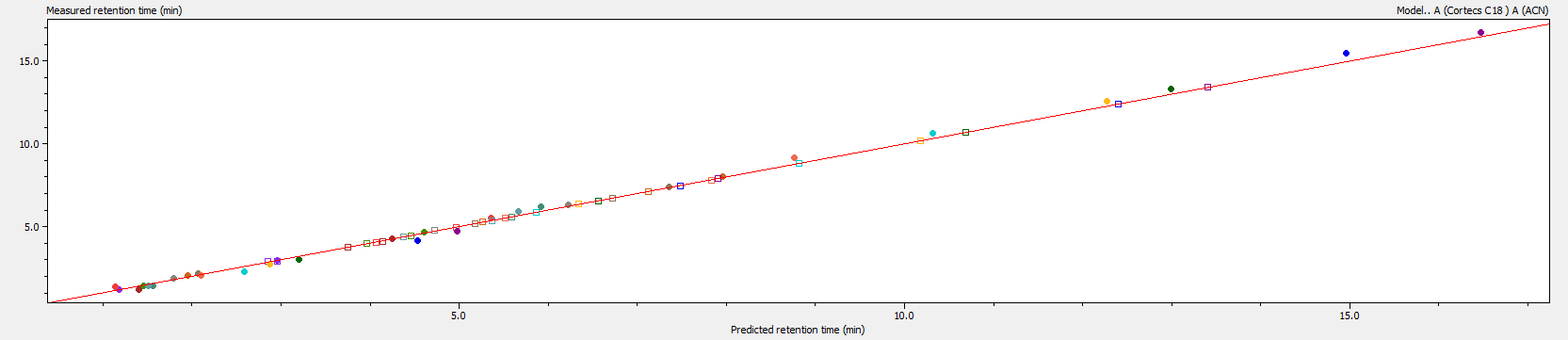

Supplement: Supplementary file 1 — (DOCX 1274 kb) [file 216_2021_3538_MOESM1_ESM.docx]
